# Fluorescent Probe as Dual-Organelle Localizer Through Differential Proton Gradients Between Lipid Droplets and Mitochondria

**DOI:** 10.1021/acs.analchem.4c01703

**Published:** 2024-05-17

**Authors:** Cinthia Hernández-Juárez, Martha Calahorra, Antonio Peña, Arturo Jiménez-Sánchez

**Affiliations:** † Instituto de Química, Universidad Nacional Autónoma de México, Ciudad Universitaria, Circuito Exterior s/n. Coyoacán 04510, Ciudad de México, México; ‡ Instituto de Fisiología Celular, Universidad Nacional Autónoma de México, Ciudad Universitaria, Circuito Exterior s/n. Coyoacán 04510, Ciudad de México, México

## Abstract

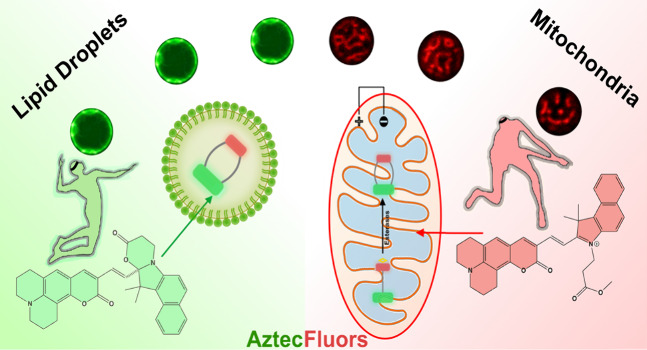

Dual-organelle molecular localizers represent powerful new tools allowing the exploration of interorganelle physical contacts and subcellular chemical communication. Here, we describe new dynamic molecular probes to localize mitochondria and lipid droplets taking advantage of the differential proton gradients present in these organelles as well as the activity of mitochondrial esterase. We unveil their potential utility when organelle retention mechanisms and proton gradients are synchronized, an insight that has not been documented previously. Our discoveries indicate that dual-organelle probes serve as a valuable multiplexing assay during starvation-induced autophagy. The pioneering molecular mechanism they employ opens doors to avoid using labile esters such as acetoxymethyl derivatives which are not optimal in imaging microscopy assays.

## Introduction

Interactions between mitochondria and lipid droplets are crucial for maintaining cellular homeostasis.^1,2^ The communication and activity of these organelles have been linked to neurodegenerative diseases via the ROS pathway.^3,4^

Lipid droplets (LDs) are dynamic organelles found in most cell types, from adipocytes to cancer cells, serve as a site of triglyceride and cholesterol storage, and are characterized by a monophospholipid membrane surrounding a hydrophobic lipid core.^5^ Crucially, the role of LDs in redox homeostasis and cellular stress has been correlated to cancer as an energy storage reservoir.^6^ This feature opens up the opportunity to connect both LDs and mitochondrial dynamics in a reliable scenario to monitor subcellular health status.

Mitochondria are essential organelles for the regulation of cellular bioenergetics and relay the switch to the glycolytic phenotype of malignant cells.^7,8^ It is known that mitochondrial function depends on its physical structure and the changes in mitochondrial morphology, which affect its network organization in response to environmental factors; such processes are known as mitochondrial dynamics.^9,10^

Of particular interest is the development of fluorescent probes to monitor the simultaneous activity and dynamics of mitochondria and LDs through imaging microscopy.^11−13^ However, using different dyes or multiple-assay techniques requires a complex workflow and separate signal recordings that may cause phenotypic drift and analytical bias.^14−16^

**Scheme 1:**
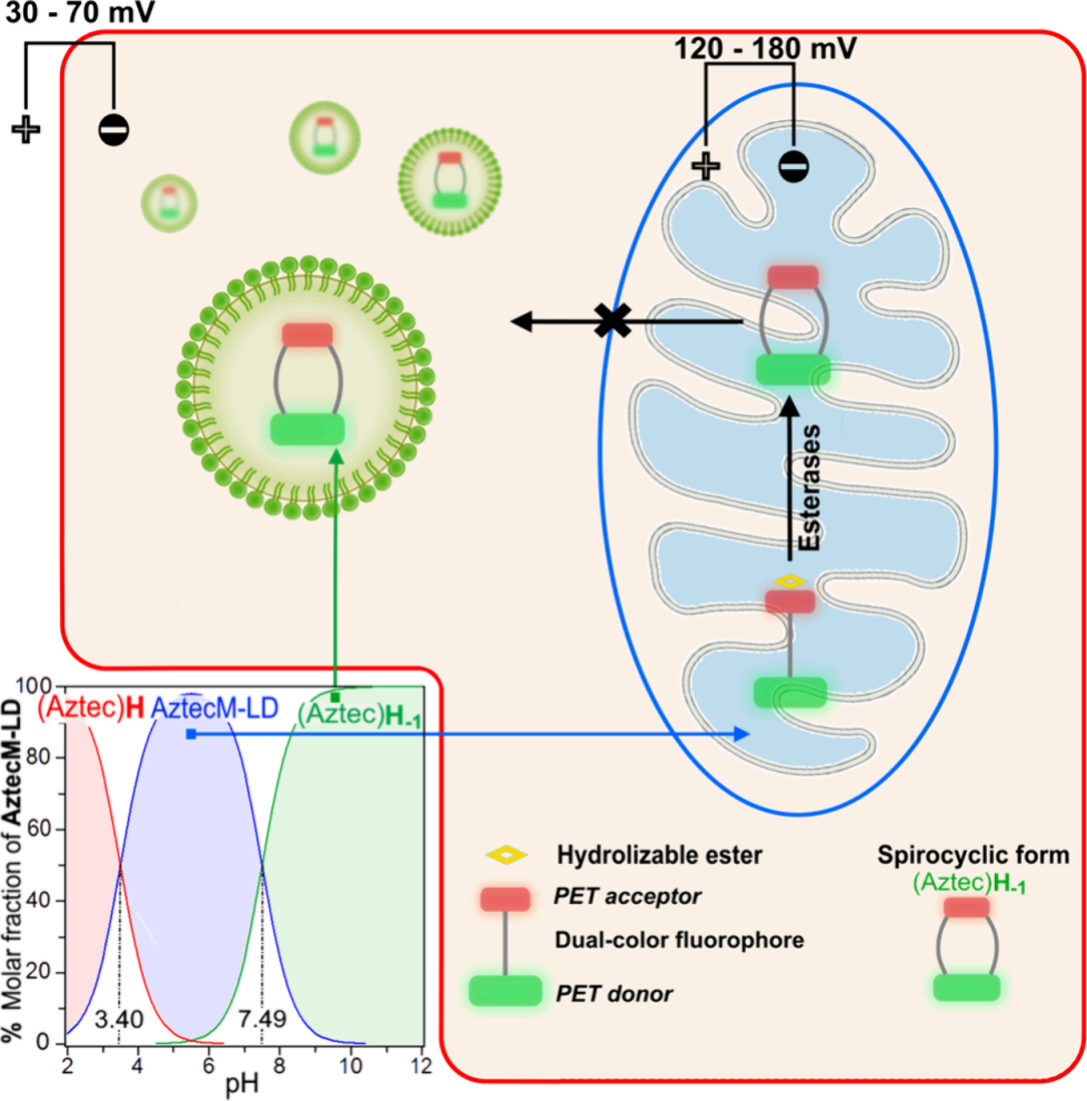
Sensing Mechanism of AztecM-LD Based on Intramolecular Spirocyclization After cytosolic esterase hydrolysis to form AztecH_–1_, LDs localization is favored, while mitochondrial esterase hydrolysis promotes mitochondrial retention since AztecH_–1_ loses its Nernstian features.

Then, our strategy to monitor mitochondria-LDs dynamics consists of the development of a new fluorescent probe called AztecM-LD, as part of our *AztecFluors* molecular family,^17,18^ able to localize mitochondria due to its Nernstian features (positively charged). After cytosol esterase hydrolysis,^19,20^ the probe favors a spirocyclic form, AztecH_–1_, changing its optical properties and localizing LDs at the green confocal channel. Also, after mitochondrial esterase hydrolysis,^21,22^ the probe is retained in mitochondria by losing its membrane potential (ΔΨ_m_) dependence, Scheme 1.

## Experimental Section

### Materials and Methods

All starting reagents were obtained from commercial suppliers and used as received.

### Synthesis

The AztecM-LD and AztecM probes were synthesized according to Scheme S1. Detailed description and chemical characterization are presented in the Supporting Information (SI) file.

### Optical Spectroscopy

The absorption spectra were acquired in a 10-mm-path-length quartz-cell Cary-50 (Varian) spectrophotometer, and the emission and excitation spectra were obtained in an Edinburgh Instruments FS500 fluorimeter at room temperature (20 ± 1 °C) under aerated conditions.

### Cell Culture, Confocal Microscopy, IC_50_ and log *P* Determination

HeLa cells as well as live human pulmonary adenocarcinoma epithelial cells (SK-Lu-1) were cultured in RPMI-1640 medium (RPMI Medium 1640 (1×), Gibco, Gaithersburg MD) supplemented with 10% fetal bovine serum (FBS, Invitrogen, Carlsbad CA), l-glutamine (2 μM), penicillin G (100 u/mL), and streptomycin sulfate (100 μg/mL) at 37 °C with 5% v/v CO_2_. SK-LU-1 cells were seeded on eight Petri dishes of 5 cm diameter with a glass bottom for 36 h before experiments using RPMI-1640 medium supplemented. Then, specific concentrations of AztecM and AztecM-LD of 1 to 8 μM were used. Commercial specific organelle localizers were added on each Petri dish 45 min before imaging experiments. All dishes were washed two times with RPMI. During confocal imaging, microscope parameters were maintained constant and excitation light was fully shielded to prevent laser artifacts. Live cells were seeded in eight-well μ-slides (iBidi, Germany) at a density of 20 000 cells per well 1 day prior to experiments in MEM alpha with 10% FBS. For lipid droplet consumption upon starvation: the culture medium from living cells preincubated with AztecM-LD was aspirated, and 1 mL of D-Hanks buffer solution was introduced. Subsequently, the cells were subjected to overnight incubation and directly imaged maintaining 5% CO_2_ and 37 °C during the experiments using an inverted Zeiss LSM 880 microscope upgraded with an incubator or a Nikon A1R upgraded with a spectral detector unit. To avoid cell autofluorescence signal contamination, laser powers were maintained at 0.05 mW (0.2% from a 25 mW laser), and untreated cells were first recorded in order to subtract any native emission signal. On treatment day for fluorescence time course experiments, cells were incubated with 1 μM of the probes for 30 min in MEM alpha with 5% FBS for the indicated time at 37 °C with 5% CO_2_, then imaged at the same conditions using 100 nM nigericin and 150 nM carbonyl cyanide *m*-chlorophenyl hydrazone (CCCP) after 5 min and 5 mg/mL oligomycin A at 20 min. For IC_50_ determination, SK-Lu-1 cells were treated with AztecM or AztecM-LD, untreated cells were considered to have 100% survival. Cell viability was determined by a redox indicator (Alamar Blue). For cytotoxicity assays, the cells were plated in 96-well plates at 5000 cells/well in RPMI-1640 medium. About 24 h after plating, varied doses of AztecM or AztecM-LD at 0.5, 1, 10, 20, 25, 35, 50, and 60 μM concentration were added in triplicate. Cell viability was evaluated after 72 h of incubation using Alamar Blue fluorescent assay (Life Technologies, Carlsbad, CA, USA). The obtained IC_50_ mean values were 91.6 ± 4.2 for AztecM and 90.5 ± 3.7 for AztecM-LD.

For yeast cell culture, two strains of yeast were used, *Saccharomyces cerevisiae* W303 (MATa/MATα {Leu2–3,112 trp1–1 can1–100 ura3–1 ade2–1 his 3–11,15} [phi^+^]) and *Saccharomyces cerevisiae* from a commercial strain (La Azteca, México); culture details can be found in the SI file. The fluorescence was monitored, and 100 μL of the 50% w/w yeast suspension was added seconds later (1), observing a small increase in the emission. In all cases, the slits of the instrument (SLM-Aminco spectrofluorometer updated by Olis) were fixed at 8 nm.

Finally, log *P* values were measured via octanol partitioning by a modification of the shake-flask method and as previously described.^23^ An aliquot of 100 mL of 300 mM of the probe in Tris buffer (10 mM, pH 7.4) and 100 mL of 1-octanol (Aldrich) were added to a 0.5 mL microtube. Buffer was employed in order to measure log *P* of the probes at physiological pH where AztecM, AztecH_–1_, and AztecM-LD exist in its neutral form. The tubes were vortexed for 1 min and centrifuged; 25 mL of each layer was removed and diluted in 100 mL of 3:1 methanol/Tris or methanol/octanol for a final composition of 3:1:1 methanol/octanol/Tris. The aqueous layer was diluted an additional 4-fold. Three dilutions were prepared per layer. A total of 100 mL of each dilution was pipetted into a 96-well plate and the absorbance read at 488 and 625 nm wavelengths. The mean *A*_500_ of three dilutions was calculated for each layer. The log (*A*_500_ of the organic layer/*A*_500_ of the aqueous layer) yielded log *P*. All absorbance measurements used were within the linear range of the instrument.

## Results and Discussion

**Figure 1:**
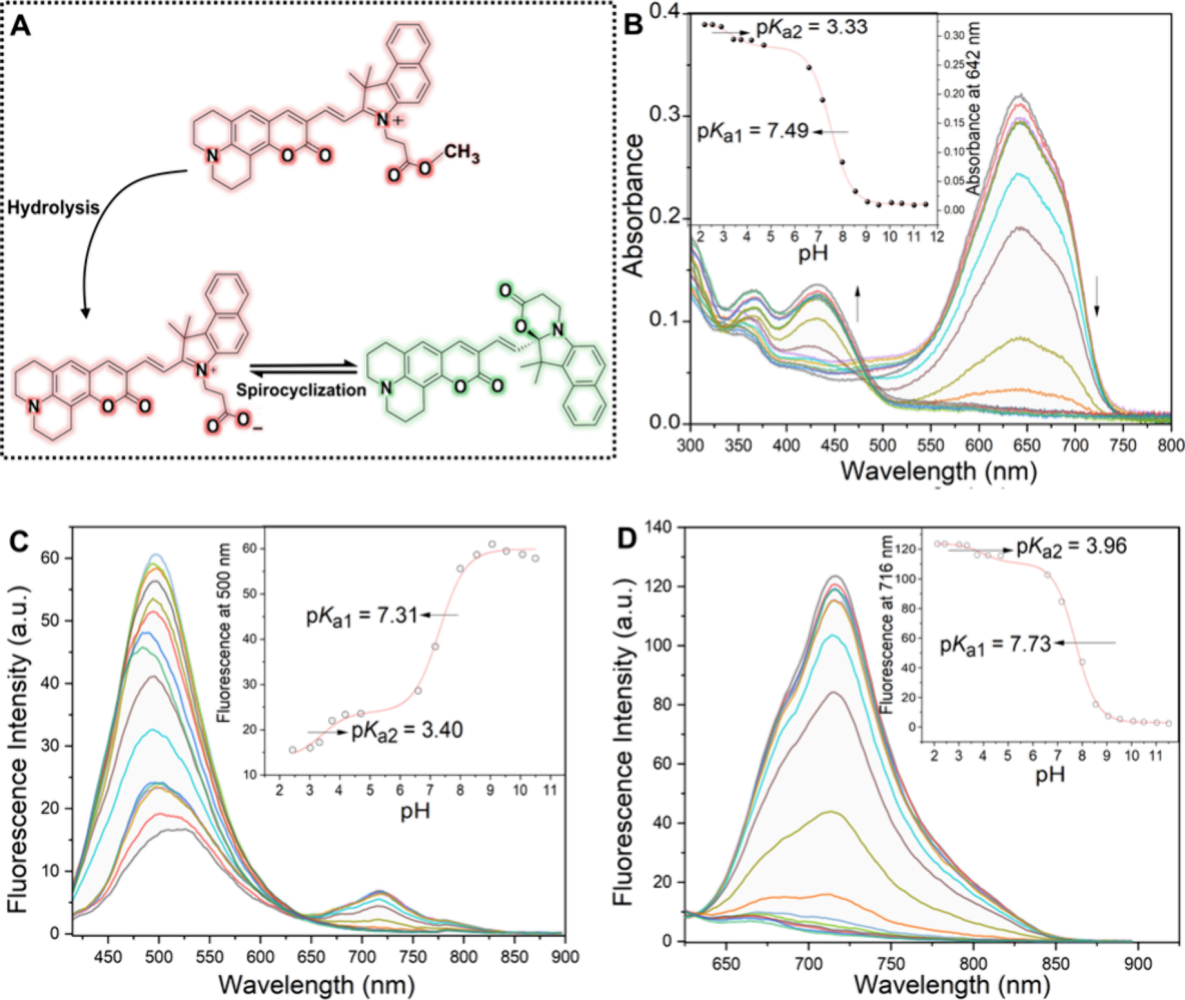
(A) Proposed hydrolysis and spirocyclization process for the AztecM-LD probe. (B) UV–vis and (C,D) fluorescence spectra of 4 μM AztecM-LD in aqueous solution. Insets show absorbance or fluorescence vs pH profiles at selected wavelengths. The excitation wavelengths are (C) 420 nm and (D) 620 nm.

**Figure 2:**
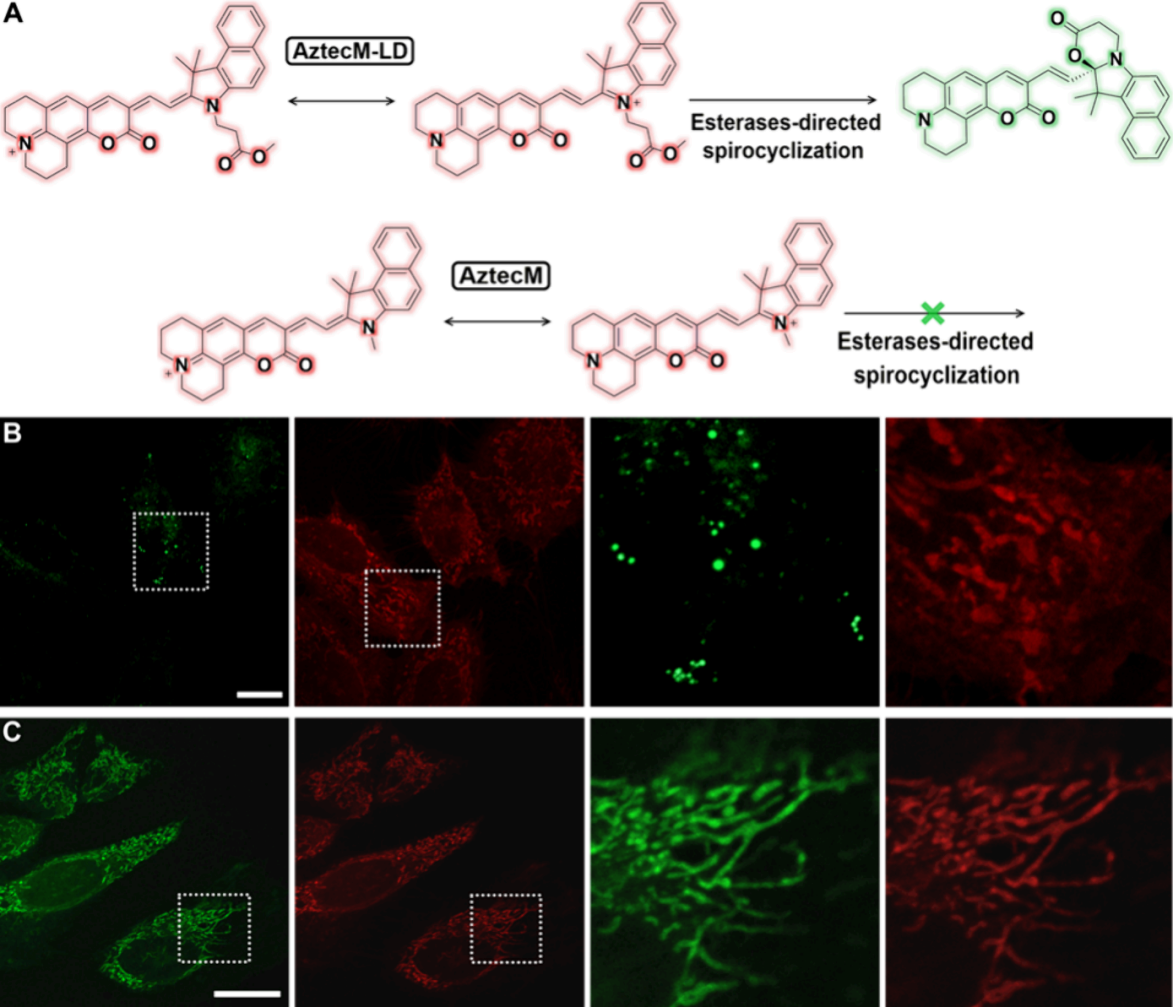
(A) Molecular structures of AztecM-LD and AztecM probes with corresponding electric-dipolar and/or spirocyclization dynamics. Confocal imaging for the localization of 4 μM (B) AztecM-LD and (C) AztecM in live SK-Lu-1 cells, detected at the green (λ_em_ = 500 nm) and red (λ_em_ = 710 nm) confocal channels. Scale bars represent 20 μm.

The probe structure is based on a coumarin-merocyanine hybrid containing an ester fragment as a hydrolyzable function upon esterase activity similar to alkanoyloxymethyl esters.^24^ Further, the lack of extensive lipophilic chains can also prevent AztecM-LD from being incorporated into lipid bilayers, thereby increasing its specificity for lipid monolayers such as LDs.^25^ Then, the photoinduced electron transfer (PET) process is modulated from the NIR fluorescence to the green coumarin 343 type emission activated after intramolecular spirocyclization, Figure 1A. Interestingly, the dual localization distribution of the open and spirocyclic forms colocalized for mitochondria and lipid droplets, respectively, Figure 2. The corresponding colocalization analysis using MitoLite Blue and NileBlue commercial dyes (Figure S15) confirmed the specific targeting ability of AztecM-LD and its hydrolyzed form AztecH_–1_, respectively.

The spectroscopic signatures of the fluorescent probes here presented display strong susceptibility to medium pH as a consequence of the spirocyclization equilibrium, Figure 2. Of note, standard pH titration conditions also promoted ester hydrolysis with subsequent cyclization, thus allowing a dual-band emission pattern through preservation of an isosbestic point at 640 nm, Figure 1C. The fitting of the titration data for UV–vis and fluorescence spectra determined p*K*_a1_ and p*K*_a2_ values as shown in Figure 1B,D insets, suggesting that the spirocyclization equilibrium (p*K*_a1_) is quite feasible in a natural subcellular environment once the AztecM-LD is hydrolyzed, *i*.e., p*K*_a1_ = 7.49 ± 0.04 (UV–vis spectra), 7.31 ± 0.05 (emission spectra, λ_exc_ = 420 nm), or 7.73 ± 0.08 (emission spectra, λ_exc_ = 620 nm).

The spectroscopic as well as the imaging profiles of this probe could not be explained with desired clarity without using a control fluorophore. Then, we next synthesized the AztecM probe (Figure 2A) in order to perform control experiments, specifically, subcellular localization features through imaging microscopy and UV–vis/fluorescence spectral profiles.

As depicted in Figure 2C, AztecM displays a filamentous-like structure that corresponds to mitochondrial distribution, the colocalization analysis using MitoTracker-Green commercial colocalizer revealed a Pearson’s coefficient of 0.92, Figure S15, SI. This is in line with a classical Nernstian behavior guided by the cationic and hydrophobic nature of the AztecM. To corroborate this mechanism, we conducted a mitochondrial ΔΨ_m_ depolarization study (*vide supra*, Figure 4). Thus, after adding the commonly used CCCP proton membrane uncoupler, we observed the fluorophore release from the mitochondrial membrane by the fluorescence signal diffusion. Overall, this strategy allowed us to conclude that the nonhydrolyzed AztecM-LD probe, which is the chemical analogue of the Nernstian AztecM probe, is able to stain mitochondria by using its membrane potential gradient. Further, the spectral profiles of AztecM and AztecM-LD display the same absorption and emission band features, indicating that no cyclization is favored, Figures S2–S4.

We then explored the imaging properties of the esterase hydrolyzed AztecM-LD probe (AztecH_–1_). As shown in Figure 2B, species AztecM-LD and AztecH_–1_ localize mitochondria under the red channel and LDs under the green channel, respectively. Then, the cyclization is promoting a neutral species with higher lipophilicity, thus driving LDs’ permeation with the stabilization of the green-emission features.

To illustrate the spirocyclic formation after hydrolysis of the AztecM-LD, the corresponding ^1^H NMR spectrum was obtained starting from the open form and confirming that the cyclization is highly favorable, see Figure S19. Also, the obtained partition coefficients (log *P*) using the standard octanol/water procedure at a constant temperature for the AztecM-LD and *in situ* hydrolyzed probe (AztecH_–1_) were 3.78 ± 0.18 and 4.82 ± 0.12, respectively. To further corroborate the ability of the closed form to prefer highly lipophilic organelles such as LDs, we also synthesized the spirocyclic derivative following lactonization under sodium ethoxide/EtOH conditions from AztecM-LD, Figure S19A, spectrum. Interestingly, we also confirmed the specificity to localize LDs in the green channel while no significant red-emission signal was detected, Figures S6 and S7. The colocalization with commercial probe monodansylpentane (MHD, #SM1000a. Abcepta, San Diego, CA) as a blue channel colocalizer is shown in Figure S20, SI.

**Figure 3:**
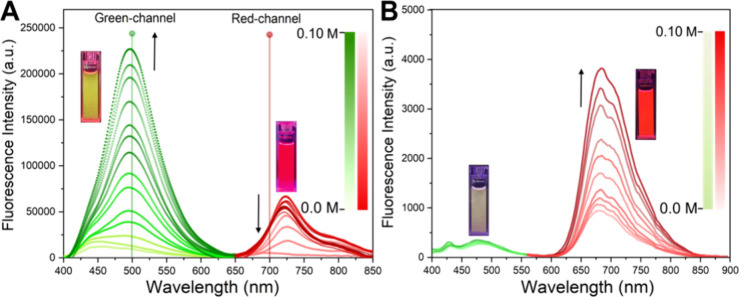
Fluorescence spectra upon increasing DOPC phospholipid concentration (0 to 0.10 M) in HEPES at pH 8 for 40 μM (A) AztecM-LD and (B) AztecM. The lines indicate green (λ_em_ = 500 nm) and red (λ_em_ = 700 nm) detection channels used in imaging microscopy. Arrows show directions of the spectral changes upon DOPC increase. Cell cuvettes show visual effects corresponding to the emission maxima.

To further corroborate if the highly lipophilic environment of LDs is promoting the AztecH_–1_ green-emission stabilization, we conducted a lipid/water titration by spectroscopic means using a 1,2-dioleoyl-*sn*-glycero-3-phosphocholine (DOPC)/H_2_O system, Figure 3. Interestingly, the spectral profiles evidenced the green-channel emission activation with a simultaneous red-band emission decrease, thus supporting the observed imaging profiles. In addition, studies for AztecM showcased a different emission pattern, where the green-channel emission remained constant while the red emission resulted in a strong activation upon DOPC concentration increments, Figure 3B. Remarkably, due to medium effects on the AztecM-LD probe, pH 8 allowed individual species distribution with significant red emission since a molar fraction of *ca*. 25% is in the open form, Scheme 1, inset. However, as medium lipophilicity increases, the red emission is not maintained, and only the green emission can be detected, Figure 3A.

Significantly, a maximum of 0.10 M DOPC concentration was upheld to simulate a uniform lipid phase devoid of liposome creation, as homogeneous lipid bilayers are not apt representations of LDs (which are inherently lipid monolayers). Thus, continuous variations studies using DOPC: H_2_O (HEPES pH 8) represent a more realistic media in terms of lipid-fluorophore interaction, supported the green-emission features.

**Figure 4:**
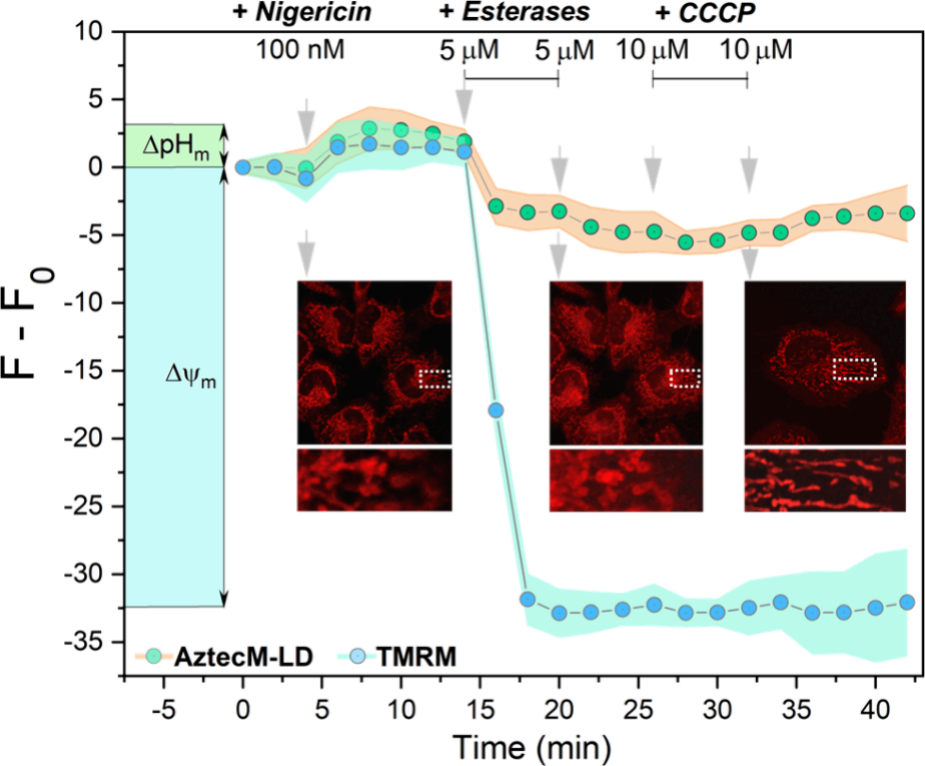
Imaging profile of SK-Lu-1 cells in the presence of 100 nM nigericin for the localization of 4 μM AztecM-LD + 10 μg/μL esterases, followed by 20 μM CCCP *in situ* additions, detected at the red (λ_em_ = 710 nm) channel. Insets show mitochondrial localization. TMRM dye profile was recorded for comparison.

Then, we aimed to elucidate the localization dynamics of AztecM-LD in live SK-Lu-1 cells by streamlining subcellular staining using the nigericin method.^26,27^ Using 100 nM nigericin, the mitochondrial pH gradient (ΔpH_m_) was effectively abolished since nigericin promotes a K^+^/H^+^ equilibration ([K^+^]_in_ = [K^+^]_out_, therefore [H^+^]_in_ = [H^+^]_out_ and ΔpH_m_ = 0), Figures 4 and S6. Thus, we stimulated the cells with exogenous esterases to hydrolyze the probe and favor LD distribution. Indeed, an LD localization was observed in the green channel with diffuse mitochondrial network staining under the red-channel setup, Figure S22. Interestingly, when a CCCP uncoupler was added to depolarize the mitochondrial potential and release any Nernstian species such as remaining AztecM-LD (open-form), no mitochondrial release was observed, indicating that the spirocycled form is retained in the organelle and do not interact with the ΔΨ_m_ gradient. This was further contrasted with the control tetramethylrhodamine (TMRM) Nernstian dye, where treatment with CCCP leads to the release of TMRM, resulting in a decrease in the fluorescence signal recordings, as shown in Figure 4. Importantly, this represents a unique alternative of exploiting the mitochondrial retention mechanism without using labile esters such as acetoxymethyl derivatives, which are not optimal for imaging studies.^28^

We then proceeded to assess the imaging scope of the probe through *in cellulo* studies conducted in yeast cells. Yeast cells are valuable tools in exploring the cell membrane potential interaction with fluorescent dyes in which intracellular pH can be finely controlled.^29,30^ As mentioned, the cell membrane potentials including plasmatic (ΔΨ_p_) or mitochondrial (ΔΨ_m_) are key factors in the transport of molecules across both membranes. Yeast cells have been used to study the interaction between the electric membrane potential and fluorescent dyes, to monitor changes in the organelle potential.^31^ This has led to a better understanding of the mechanisms involved in the transport of molecules across cellular membranes. By studying the behavior of these cargoes in yeast cells, further insights can be gained into the mechanisms that regulate the transport and retention of molecules in the mitochondrial membrane.^32^

**Figure 5:**
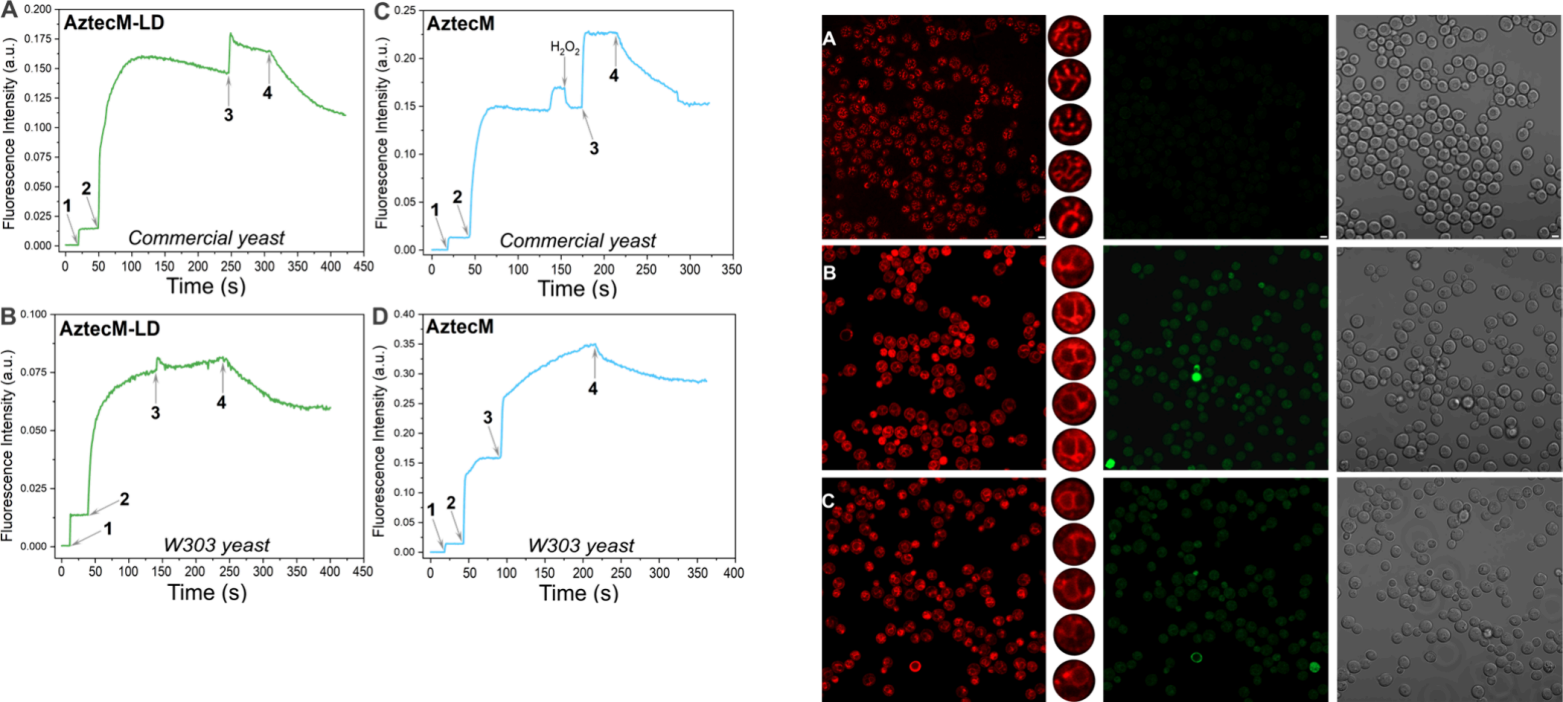
Fluorescence variation effect of *Saccharomyces cerevisiae*: Panels A and B are commercial yeast while C and D are W303 yeast, stained with AztecM-LD and AztecM probes. (1) Yeast addition; (2) 2 μM fluorophore addition; (3) addition of CCCP uncoupler; and (4) addition of 20 mM KCl. λ_em_ = 500 nm, arrows indicate addition intervals.

Thus, two strains of yeast were used, *Saccharomyces cerevisiae* W303 (MATa/MATα {Leu2–3,112 trp1–1 can1–100 ura3–1 ade2–1 his 3–11,15} [phi^+^]) and *Saccharomyces cerevisiae* from a commercial strain (La Azteca, México). The obtained graphs for probes AztecM-LD and AztecM are shown in Figure 5. As detailed in the experimental section, the fluorescence was monitored upon 100 μL of the 50% w/w yeast suspension addition (1), observing a small fluorescence increment. Once this emission was constant, 2 μM fluorophore was added (2) and an increase in emission observed due to the accumulation of the probe in the cytoplasm and mitochondria. However, the accumulation of the probe in the mitochondria is such that a partial quenching of the fluorescence takes place. Then, the addition of 10 μM CCCP (label 3), which depolarizes the mitochondrial membrane potential, produced a large increase of the fluorescence, because the probe was released into the cytoplasm.

**Figure 6:**
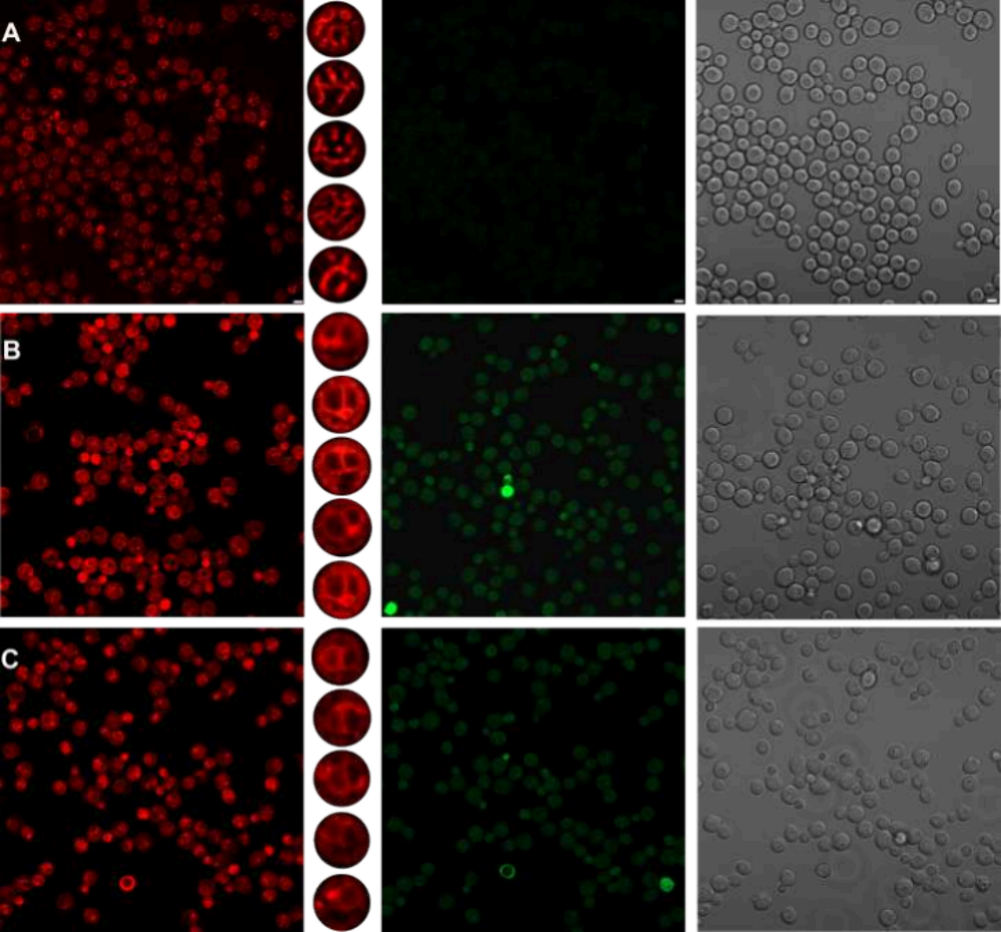
Imaging microscopy of *Saccharomyces cerevisiae* commercial yeast stained with (A) 2 μM AztecM-LD, (B) 10 μM CCCP uncoupler, and (C) KCl stimuli. Red-channel insets depict selected facial-like shapes illustrating the ability of the probe to track (A) membrane dynamics or (B,C) membrane localization, detected at the green (λ_em_ = 500 nm) and red (λ_em_ = 710 nm) confocal channels. Scale bars represent 5 μm.

It is important to note that the dye does not enter the vacuole, which has a positive membrane potential (Figure 6 and Figure S23, SI). This effect was more clearly observed for AztecM, which is mainly accumulated by the mitochondria, but also in the cytoplasm, due to the electric membrane potential of each one. A minor effect was observed for AztecM-LD in commercial yeast or no effect in W303 yeast, which is attributed to the ester group hydrolysis and subsequent mitochondrial retention of the fluorophore due to the total charge neutralization. Then, upon adding potassium ions (20 mM KCl, label 4), it enters through its transporters Trk1 and Trk2, partially reducing the potential difference of the plasma membrane. That is why part of the probe leaks out and, consequently, the fluorescence decreases. This effect was more significant for AztecM, commercial yeast.

Additionally, since commercial yeast presents faster cellular respiration than the W303 strain, and therefore medium oxygen is consumed more quickly, the addition of H_2_O_2_ (5 μL of a 3% aqueous solution) was performed when an increase in emission was observed after stabilizing the signal following the fluorophore addition. This was only necessary for the experiment performed with AztecM, since the decrease in the mitochondrial membrane potential due to oxygen depletion causes the fluorophore to diffuse into the medium, leading to an increase in the emission signal. After the addition of H_2_O_2_, the emission signal decreased and remained at a similar level to the previously observed signal. This increase in emission was not observed in AztecM-LD, suggesting the activation of the mitochondrial retention mechanism. Finally, by the addition of KCl the depolarization of the plasma membrane potential is observed.

The imaging microscopy studies showed variations between the commercial and W303 yeasts, indicating differences in mitochondrial quantity and dynamics. However, the fluorescence localization pattern remained consistent. Figure 6 displays the localization images obtained for the AztecM-LD probe in the commercial yeast, while the corresponding imaging for the W303 yeast can be found in Figure S23, SI. The observed fluorescence distribution patterns suggest that initially, the probe strongly localizes in the mitochondria. However, after CCCP stimuli, the probe fluorescence signal redistributes to the plasma membrane region, because of the mitochondrial depolarization, although in the green channel a residual mitochondrial localization persisted, indicating a small fraction of the spirocyclic form of AztecM-LD, similar to the mitochondrial distribution pattern showed in Figure 4B–D, yellow arrows in the second column. Subsequently, the addition of KCl leads to a subtle decrease in the fluorescence signal and distribution. Detailed Z-scans and continuous monitoring of the AztecM-LD probe over time can be found in Movie S1.

The results obtained with the AztecM probe aligned with the anticipated outcomes. Following CCCP-induced depolarization, the probe localization changed from the mitochondria to the plasma membrane compartment. Subsequently, upon KCl stimulation, a notable decrease in fluorescence intensity was observed. Importantly, imaging microscopy results for W303 yeast indicated a consistent fluorescence localization pattern, see Figure S23.

**Figure 7:**
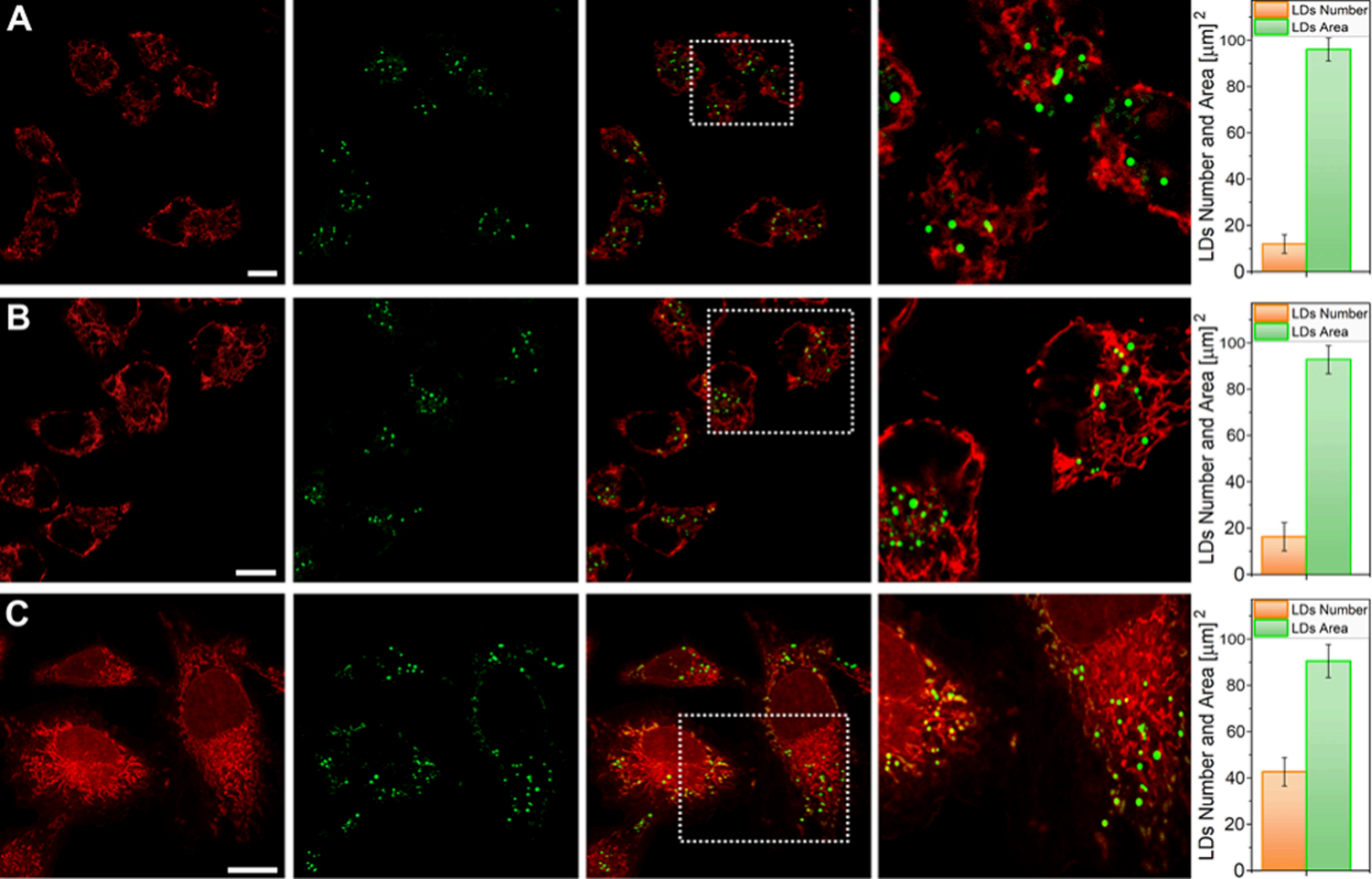
High-resolution confocal imaging using the Airyscan technique for the localization of 4 μM AztecM-LD in live SK-Lu-1 cells, detected at the green (λ_em_ = 500 nm) and red (λ_em_ = 710 nm) confocal channels. Panels A and B depict images obtained using nigericin to achieve pH values of 4.5 and 8.5, respectively, while panel C displays imaging under conditions of starvation-induced LD consumption. Panels on the right show LD counting (orange bars) vs LD average area in μm^2^ (green bars). Scale bars represent 20 μm.

Finally, the study of interorganellar interaction is an emerging field in cell biology that appears to be crucial for cellular adaptation to nutrient availability.^33^ Consequently, we developed a model to simulate LD consumption and expansion guided by pH gradients during cell starvation. To effectively monitor LD consumption and expansion, a dual-targeted fluorescent probe able to be retained in both mitochondria and LDs during variations in mitochondrial potential depolarization/hyperpolarization is highly desirable. This is because, following LD expansion, mitochondrial dynamics lead to the separation of mitochondria into cytosolic and peri-droplet compartments.^34^ Therefore, the AztecM-LD probe can accurately track dynamic changes during these expansion and contraction processes. Figure 7 shows the AztecM-LD localization in LDs and mitochondria in live SK-Lu-1 cells conducted under conditions where the pH was adjusted using nigericin to achieve pH values of 4.5 and 8.5. This was done in a high-K^+^ buffer during the incubation period and in D-Hanks cell culture medium for starvation-induced LD consumption.

As depicted in Figure 7, following 12-h incubation with oleic acid to increase the population of LDs, there was an increment in the number of LDs at pH 4.5, while only a slight additional increase was observed at pH 8.5. As shown in Figure 7C, under conditions of starvation-induced autophagy, there was notable fragmentation or ripening of lipid droplets, resulting in an increase in the quantity of these organelles, despite no significant change was observed in LD size, Figure 7 (right panels); this is in agreement with previous findings where during starvation the LDs are not necessarily decreasing but maintaining or even increasing.^35^ Here, we observed that this redistribution of lipid droplets is directly linked to mitochondrial dynamics and could be involved as a protective lipid system in cellular stress responses, such as starvation.

## Conclusions

Fluorescent dyes have become an essential tool for imaging microscopy, offering detailed insights into cellular processes. The dual-organelle localization of AztecM-LD type probes represents a promising approach for simultaneously investigating multiple cellular structures and functions. Nonetheless, the demand for more efficient and selective molecular probes capable of targeting and staining other organelles persists. In response, we have developed a dual-organelle targeted fluorescent probe with pH-driven cellular localization, serving as a potent tool for imaging both mitochondria and lipid droplets. The successful application of the AztecM-LD probe enabled the monitoring of dynamic changes during LD expansion and contraction processes induced by significant pH variations and under conditions of starvation-induced autophagy, revealing notable fragmentation of LDs. Importantly, this provides a unique strategy to leverage the mitochondrial retention mechanism, eliminating the necessity for less appropriate labile esters such as acetoxymethyl derivatives in imaging studies.

## Supporting Information Available

Probes synthesis data, UV–vis and fluorescence spectroscopy and lifetime measurements, cell culture and imaging colocalization data (PDF)
Yeast mitochondrial dynamics (AVI)

## Supplementary Material

ac4c01703_si_001.pdf

ac4c01703_si_002.avi
