# Supplementary material for: Fluorescent Probe as Dual-Organelle Localizer Through Differential Proton Gradients Between Lipid Droplets and Mitochondria
Source: Anal Chem. 2024 May 17;96(22):9262–9. doi: 10.1021/acs.analchem.4c01703 (PMC11154735; doi:10.1021/acs.analchem.4c01703)
Supplement: Supplementary file 1 — ac4c01703_si_001.pdf [file ac4c01703_si_001.pdf]

Supporting Information

Fluorescent Probe as Dual-Organelle Localizer Through Differential Proton Gradients Between Lipid Droplets and Mitochondria

Cinthia Hernández-Juárez,[a] Martha Calahorra,[b] Antonio Peña,[b] and Arturo Jiménez-Sánchez*,[a]

[a] Bioorganic Chemistry Laboratory (BioCheLa) at Instituto de Química and

[b] Instituto de Fisiología Celular. Universidad Nacional Autónoma de México (UNAM). Circuito Exterior s/n, Coyoacán 04510. Ciudad Universitaria, Ciudad de México, México. E-mail: arturo.jimenez@iquimica.unam.mx

Table of Contents

Experimental procedures **S3**

1) Materials, physical measurements, and cell culture methods **S3**

2) Determination of fluorescence quantum yields **S3**

3) Cell culture, confocal microscopy and IC50 /log P determination **S4**

4) Yeast cells maintenance, imaging and Table S1 **S5**

5) Compound synthesis and chemical characterization **S6**

Acknowledgements **S31**

Experimental Procedures

Materials, physical measurements, and cell culture methods

Commercially available starting materials, components of buffer solutions (CHES, MOPS, MES from Sigma, Mexico) and solvents were used as supplied. ^1H and ^{13}C NMR spectra were recorded at room temperature on a 500 MHz Bruker unity spectrometer. Chemical shifts (ppm) are relative to $(CH_3)_4Si$. High resolution mass spectrometry (ESI-TOF) was obtained by using an Agilent Technologies 6530 Accurate-Mass Q-TOF LC/MS equipment. Fluorescence experiments were measured either on a FS5 spectrofluorometer from Edinburgh Instruments or in a Cary Eclipse fluorimeter from Agilent, UV-Vis absorption spectra were taken on a Thermo Scientific Evolution diode array UV-Vis spectrophotometer. For light irradiation sources for the photochemical reactions we used both, xenon lamp 300 W irradiation or LED-based portable light for PDT VL400-EMITTER and LED PAR38 lamp (19W, 2700K, 1380 lumen).

Determination of the fluorescence quantum yield

Fluorescence quantum yield for **AztecM** of 0.32 and **AztecM-LD** of 0.41 were determined by using Rhodamine B ($\phi_F = 0.490$ in ethanol)[1] as a fluorescence standard. The quantum yield was calculated using the following equation (S1):

$$\phi_{F(X)} = \phi_{F(S)}(A_S F_X / A_X F_S)(n_X / n_S)^2 \qquad \text{eq. (S1)}$$

where ϕ_F is the fluorescence quantum yield, A is the absorbance at the excitation wavelength, F is the area under the corrected emission curve, and n is the refractive index of the solvents used. Subscripts S an X refer to the standard and to the unknown, respectively.

[1] Rurack, K. and Spieles, M. Fluorescence Quatum Yields of a Series of Red and Near-Infrered Dyes Emittingat 600-1000 nm. *Anal. Chem.* 2011, 83, 4, 1232-1242. https://doi.org/10.1021/ac101329h

Cell Culture, Confocal Microscopy, IC_{50} and logP determination. HeLa cells as well as live human pulmonary adenocarcinoma epithelial cells (SK-Lu-1) were cultured in RPMI-1640 medium (RPMI Medium 1640 (1x), Gibco, Gaithersburg MD) supplemented with 10% fetal bovine serum (FBS, Invitrogen, Carlsbad CA), L- glutamine (2 μM), penicillin G (100 u/mL), streptomycin sulfate (100 μg/ mL) at 37°C with 5% v/v CO_2. Live SK-LU-1 cells were seeded on 8 Petri dishes of 5 cm diameter with glass bottom for 36 hours before experiments using RPMI-1640 medium supplemented. Then, specific concentrations of **AztecM** and **AztecM-LD** 1 to 8 μM were used. Commercial specific organelle localizers were added on each Petri dish 45 minutes before imaging experiments. All dishes were washed two times with RPMI. During confocal imaging, microscope parameters were maintained constant and excitation light was fully-shielded to prevent laser artefacts. Live cells were seeded in 8 well μ–slides (iBidi, Germany) at a density of 20000 cells per well one day prior to experiments in MEM alpha with 10% FBS. On treatment day, cells were washed once in MEM alpha with no FBS and incubated with 1 to 8 μM **AztecM** and **AztecM-LD** probes for 30 minutes. For experiments with MitoLiteBlue®, 50 nM were added 10 minutes before **AztecM** and **AztecM-LD**. Cells were then washed twice in MEM alpha with no FBS and imaged maintaining 5% CO_2 and 37°C during the experiments using an inverted Zeiss LSM 880 microscope upgraded with an incubator or a Nikon A1R upgraded with a spectral detector unit. To avoid cell autofluorescence signal contamination, laser powers were maintained at 0.05 mW (0.2% from a 25 mW laser) and untreated cells were first recorded in order to subtract any native emission signal. On treatment day for fluorescence time course experiments, cells were incubated with 1 μM of the probes for 30 minutes in MEM alpha with 5% FBS for the indicated time at 37°C with 5% CO_2, then imaged at the same conditions using 100nM nigericin, 150 nM CCCP (after 5 min) and 5 mg/mL oligomycin A at 20 min. For IC_{50} determination, SK-Lu-1 cells were treated with **AztecM** or **AztecM-LD**, untreated cells were considered to have 100% survival. Cell viability was determined by a redox indicator (Alamar Blue). For cytotoxicity assays, the cells were plated in 96-well plates at 5000 cells/well in RPMI-1640 medium. About 24 h after plating, varied doses of **AztecM** or **AztecM-LD** at 0.5, 1, 10, 20, 25, 35, 50 and 60 μM concentration were added in triplicate. Cell viability was evaluated after 72-h incubation using Alamar Blue fluorescent assay (Life Technologies, Carlsbad, CA, USA). The obtained IC_{50} mean values were 91.6 ± 4.2 for **AztecM** and 90.5 ± 3.7 for **AztecM-LD**.

Finally, Log *P* values were measured via octanol partitioning by a modification of the shake-flask method and as previously described (reference #5 main text). An aliquot of 100 ml of 300 mM of the probe in Tris buffer (10 mM, pH 7.4) and 100 ml 1-octanol (Aldrich) were added to a 0.5 ml microtube. Buffer was employed in order to measure log *P* of the probes at physiological pH where **AztecM**, **AztecH$_{-1}$**and **AztecM-LD** exist in its neutral form. The tubes were vortexed for 1 min and centrifuged; 25 ml of each layer was removed and diluted in 100 ml 3:1 methanol:Tris or methanol:octanol for a final composition of 3:1:1 methanol:octanol:Tris. The aqueous layer was diluted an additional 4-fold. Three dilutions were prepared per layer, 100 ml of each dilution was pipetted into a 96 well plate, and the absorbance read at 488 nm and 625 nm wavelengths. The mean A_{500} of three dilutions was calculated for each layer. The log (A_{500} of the organic layer/A_{500} of the aqueous layer) yielded log *P*. All absorbance measurements used were within the linear range of the instrument.

Table S1. Relevant physicochemical characterization values for the Aztec Fluors probes.

Probe	Log *P* [a]	ϕ_F [b]	τ_{em} [c]	IC_{50}
**AztecM**	4.05 ± 0.21	0.32	57 ± 0.2 ps	91.6 ± 4.2
**AztecM-LD**	3.78 ± 0.18	0.41	288 ± 5 ps	90.5 ± 3.7
**AztecH$_{-1}$**	4.82 ± 0.12	n.d.	n.d.	n.d.

[a] Partition coefficient. [b] fluorescent quantum yield. [c] Fluorescence lifetimes τ_1 in picoseconds obtained by time-correlated single photon counting technique (TCSPC) recorded in DMSO. Notes: n.d. (not determined).

Yeast cells maintenance and imaging

Two strains of yeast were used, *Saccharomyces cerevisiae* W303 (*MATα/MATα {Leu2-3,112 trp1-1 can1-100 ura3-1 ade2-1 his 3-11,15} [phi$^+$]*) and *Saccharomyces cerevisiae* from a commercial strain (*La Azteca, México*). After obtaining the culture in YPD media at 30 °C, it was left fasting for 24 hours, then a 50% aqueous suspension was prepared by weight, and a temporal monitoring of fluorescence emission was carried out by adding different solutions to a stock mixture (1.83 mL of 0.1M MES-TEA buffer, 2 µL of 10 mM $BaCl_2$, and 40 µL of 1M glucose) at pH 6, further culture details can be found in the ESI file.

Compound synthesis and chemical characterization

Scheme S1 describes the synthetic methodology followed to prepare fluorophores.

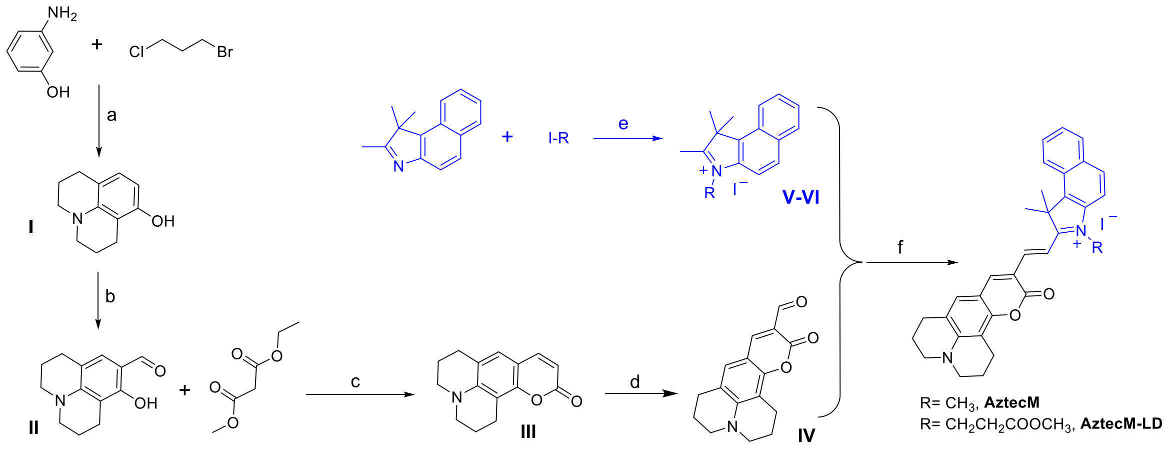

Scheme S1. General synthetic methodology to obtain molecules I-VI and fluorescent probes. Reagent conditions: a: $NaHCO_3$ (3.6 equiv), dry DMF, heat 90°C; b: 0-5 °C, DMF (2.4 equiv), $POCl_3$ (1.6 equiv), rt 1 h, reflux, 1 h; c: EtOH, piperidine (1 equiv), reflux, 24 h, concentrated HCl/acetic acid, heat 80°C 12 h, rt, NaOH solution 20%; d: 0-5 °C, DMF (2.4 equiv), $POCl_3$ (1.6 equiv), rt 1 h, reflux, overnight; e: Acetonitrile, reflux 24 or 48 h; f: ethanol, reflux 12 h.

Synthesis of I (8-Hydroxyjulolidine)

3-aminophenol (5 g, 45.82 mmol), 1-bromo-3-chloropropane (15 mL, 151.49 mmol) and $NaHCO_3$ (14 g, 166.65 mmol) were dissolved in 30 mL of anhydrous DMF. The mixture was stirred and heated at 90 °C for 24 hours. It was allowed to cool and AcOEt: H_2O extraction was performed. The organic phase was recovered and purified by column chromatography (hexane:AcOEt). A white solid was obtained. Yield: 35%.

^{1}H NMR (400 MHz, ($CDCl_3$) δ in ppm: 6.66 (d, *J*=7.98 Hz, 1H), 6.05 (d, *J*=7.98 Hz, 1H), 3.12-3.07 (m, 4H), 2.73-2.62 (m, 4H), 2.03-1.93 (m, 4H).

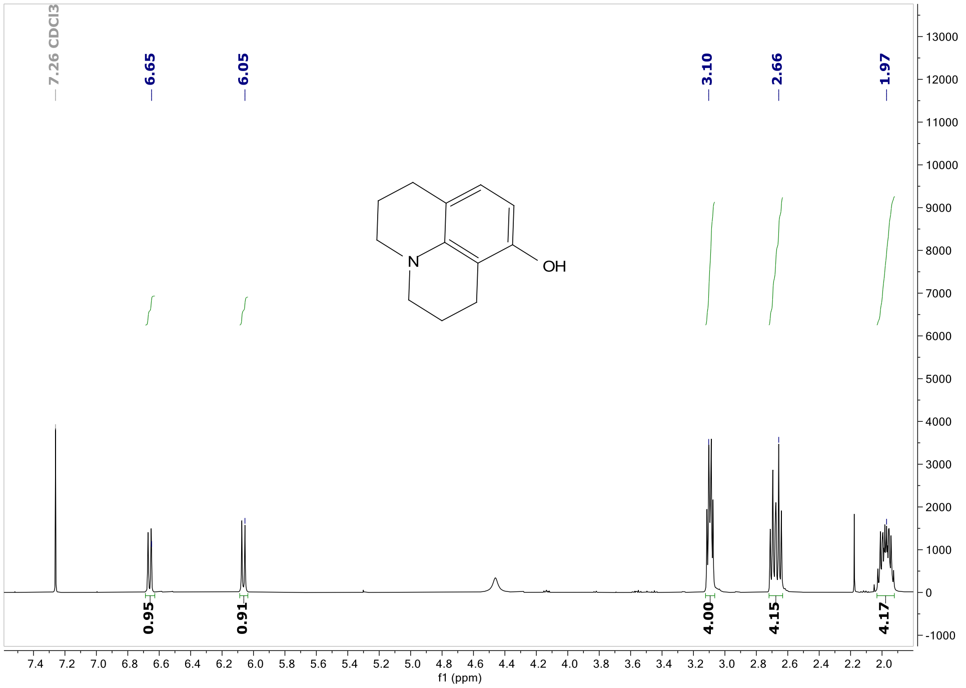

Figure S1. ^{1}H NMR spectrum (400 MHz, $CDCl_3$) of compound **I**.

Synthesis of II (8-Hydroxyjulolidine-9-carboxaldehyde)

In an ice bath, 2.4 mL of $POCl_3$ (25.67 mmol) were added dropwise to 3 mL of DMF (38.75 mmol), this mixture was left stirring for 30 min. The compound **I** (3.03 g, 16 mmol) was dissolved in 4 mL of DMF and added to the previous mixture keeping the ice bath, the new mixture was stirred for another 30 min at room temperature. The mixture was refluxed for one hour and subsequently allowed to cool. 200 g of ice were added and was left stirring until the formation of a precipitate was observed. It was filtered and allowed to dry to obtain a blue-green solid which was thus used for the next reaction. Yield: 90%

Synthesis of III (2,3,6,7-Tetrahydro-1H,5H,11H-[1]benzopyrano[6,7,8-*ij*]quinolizin-11-one)

Compound **II** (2.9 g, 13.34 mmol) and diethyl malonate (4.23 mL, 30.39 mmol) were dissolved into 20 mL ethanol, then 1.27 mL piperidine (13.66 mmol) was added. The mixture was stirred and refluxed for 24h. After that, the solvent was removed by vacuum to obtain a solid. Subsequently, 25 mL of concentrated hydrochloric acid (295.9 mmol) and 20 mL glacial acetic acid (349.7 mmol) were added sequentially to a flask with the solid. Then mixture was stirred and heated to 80 ℃ for 12 h. After the reaction was accomplished, the solution was cooled to room temperature. The pH of the solution was adjusted to 7.0 after pouring the solution into 400 mL ice water and adding 20 % NaOH solution. Yellow precipitate slowly appeared in the solution along with stirring for 30 min. Afterwards, the mixture was filtered, washed with pure water and a yellow precipitated was obtained. Yield: 62%.

^{1}H NMR (400 MHz, ($CDCl_3$) δ in ppm: 7.44 (d, *J*=9.24 Hz, 1H), 6.84 (s, 1H), 5.97 (d, *J*=9.24 Hz, 1H), 3.26 (q, *J*=5.83 Hz, 4H), 2.88 (t, *J*= 6.51 Hz, 2H), 2.75 (td, *J*= 6.36, 1.07 Hz, 2H), 2.02-1.91 (m, 4H).

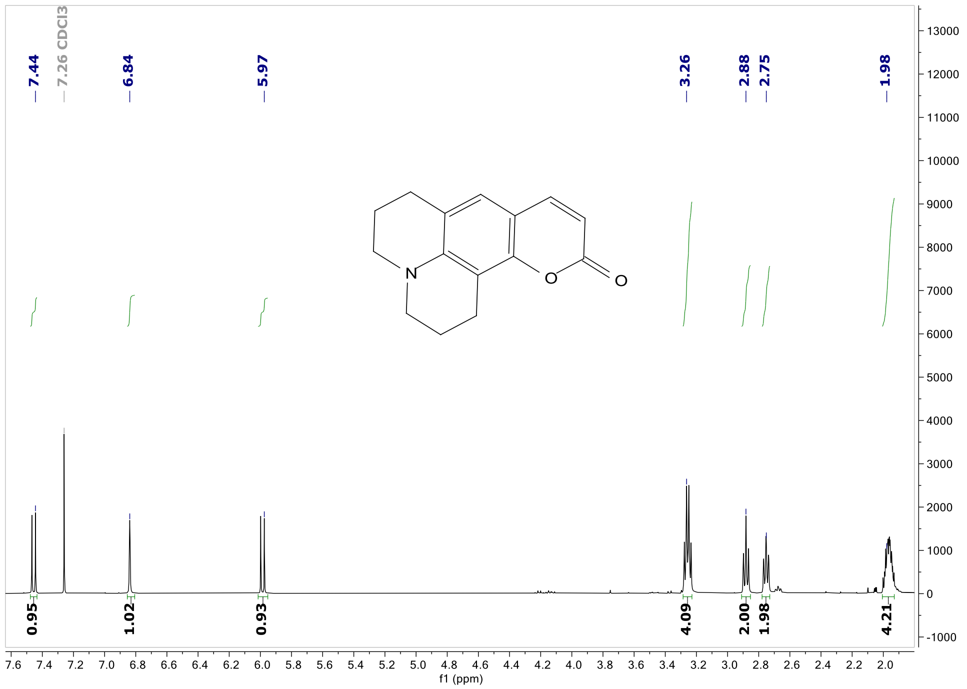

Figure S2. ^{1}H NMR spectrum (400 MHz, $CDCl_3$) of compound **III**.

Synthesis of IV (11-Oxo-2,3,6,7-tetrahydro-1H,5H,11H-pyrano[2,3-f]pyrido[3,2,1-*ij*]quinoline-10-carbaldehyde)

Compound **IV** was done following the same procedure of compound **II** but modifying the reflux time (overnight) and the product was purified by chromatography column with hexane:AcOEt 1:1. An orange solid was obtained. Yield: 45%.

Synthesis of V (1,1,2,3-tetramethyl-1*H*-benzo[*e*]indol-3-ium) and VI (3-(3-methoxy-3-oxopropyl)-1,1,2-trimethyl-1*H*-benzo[*e*]indol-3-ium)

Compound **V** was obtained by quaternization reaction of 1,1,2-trimethylbenz[e]indole. 1,1,2-trimethylbenz[e]indole (500 mg, 2.38 mmol) and iodomethane (220 µL, 3.53 mmol) were dissolved in acetonitrile. The mixture was stirred and refluxed for 24 h. The solvent was evaporated under reduced pressure. The residue was dissolved in a minimum amount of methanol, then cold ethyl acetate was added. The precipitate was separated by filtration, washed with ethyl acetate and acetone, then the solid was dried. A light-yellow solid was obtained. Yield: 80%

Compound **VI** was synthesized following the previous procedure but using methyl 3-iodopropionate (750 mg, 3.50 mmol) and the mixture was refluxed for 48 h, other steps were the same. A light blue solid was obtained. Yield: 73%.

Synthesis of probes AztecM and AztecM-LD

Fluorescent probes were carried out using the same methodology and only changing the quaternary salt. Compound **IV** (100 mg, 0.37 mmol) were dissolved in 10 mL of ethanol, 1.5 equivalents of the corresponding salt (**V** or **VI**) and piperidine (10 µL, 0.1 mmol) were added. The mixture was refluxed for 12 hours. The products were purified by column chromatography using DCM:MeOH as eluent

(5% methanol). An iridescent green solid (yield: 65%) and an iridescent dark purple solid was obtained for **AztecM-LD** (yield: 35%).

AztecM ^{1}H NMR (400 MHz, (DMSO-d_6) δ in ppm: 8.69 (s, 1H), 8.4 (d, *J*=8.53 Hz, 1H), 8.34 (d, *J*=15.83 Hz, 1H), 8.24 (d, *J*=8.92 Hz, 1H), 8.18 (d, *J*=7.54 Hz, 1H), 8.03 (d, *J*=8.92 Hz, 1H), 7.84-7.74 (m, 2H), 7.68 (ddd, *J*=8.05, 6.86, 1.09 Hz, 1H), 4.06 (s, 3H), 3.47-3.43 (m, 4H), 2.78 (t, *J*=6.15 Hz, 4H), 1.98 (s, 6H), 1.96-1.87 (m, 3H). ^{13}C NMR (100 MHz, $CDCl_3$) δ in ppm: 181.84, 161.46, 153.67, 151.06, 150.12, 149.0, 139.11, 137.13, 133.25, 131.42, 130.34, 130.16, 128.53, 127.73, 126.86, 122.78, 121.05, 111.72, 111.43, 107.64, 106.14, 53.57, 51.17, 50.60, 36.11, 27.5, 27.26, 21.09, 20.12, 19.96. ESI HPLCMS m/z= 475.2320 $[M]^+$ found, calculated m/z= 415.24.

AztecM-LD ^{1}H NMR (400 MHz, (DMSO-d_6) δ in ppm: 8.68 (s, 1H), 8.44-8.35 (m, 2H), 8.22 (d, *J* = 8.91 Hz, 1H), 8.17 (d, *J* = 7.80 Hz, 1H), 8.04 (d, *J* = 8.99 Hz, 1H), 7.80 (d, *J* = 15.71 Hz, 1H), 7.79 – 7.74 (m, 1H), 7.67 (t, *J* = 7.51 Hz, 1H), 7.20 (s, 1H), 4.79 (t, *J* = 6.90 Hz, 2H), 3.57 (s, 3H), 3.48-3.46 (m, 4H), 3.07-3.04 (m, 2H), 2.78 (t, *J* = 6.28 Hz, 4H), 1.99 (s, 6H), 1.91 (q, *J* = 8.02, 6.72 Hz, 4H). ^{13}C NMR (100 MHz, $CDCl_3$) δ in ppm: 181.55, 170.81, 161.53, 153.87, 151.43, 138.48, 137.18, 133.14, 131.70, 131.18, 130.45, 130.30, 128.77, 128.42, 127.75, 126.80, 122.74, 121.22, 111.95, 111.78, 111.66, 107.08, 106.27, 53.12, 52.37, 51.28, 50.70, 43.04, 33.38, 27.88, 27.23, 21.08, 20.11, 19.93. ESI HPLCMS m/z= 547.2510 $[M]^+$ found, calculated m/z= 547.26.

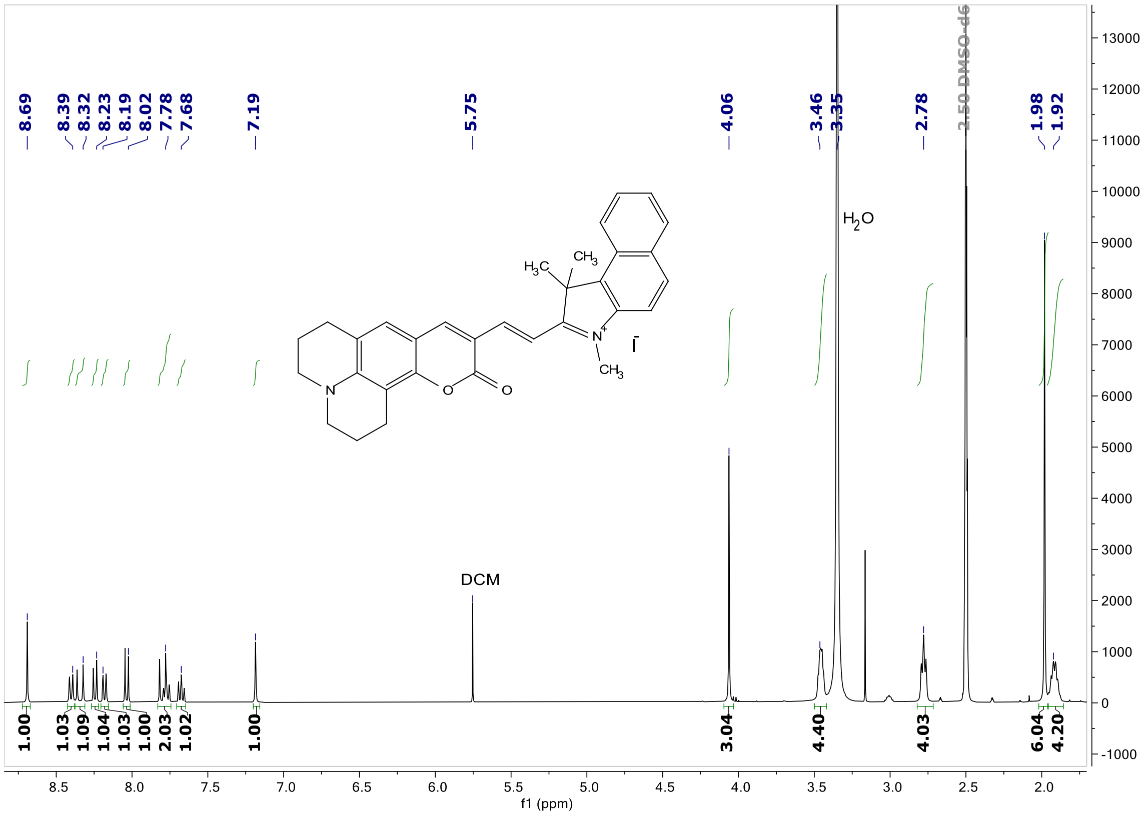

Figure S3. ^{1}H NMR spectrum (400 MHz, DMSO-d_6) of **AztecM**.

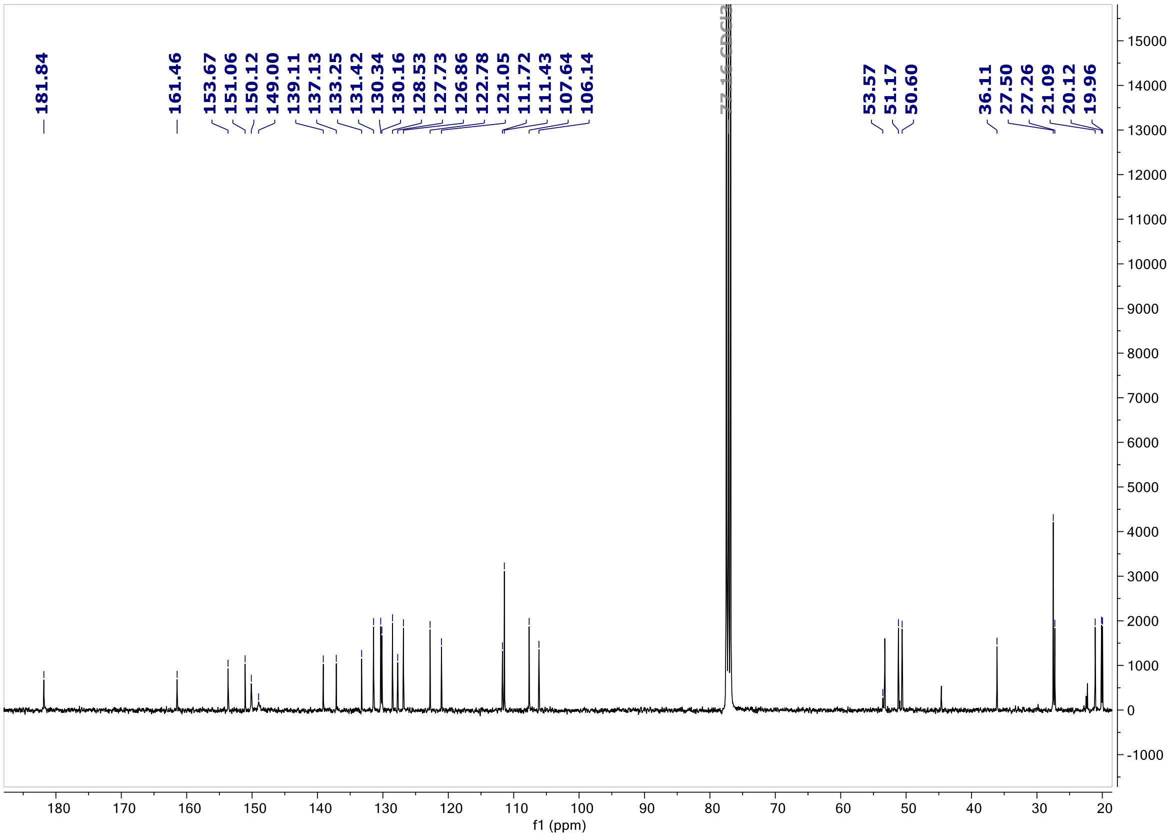

Figure S4. ^{13}C NMR spectrum (101 MHz, in $CDCl_3$) of **AztecM**.

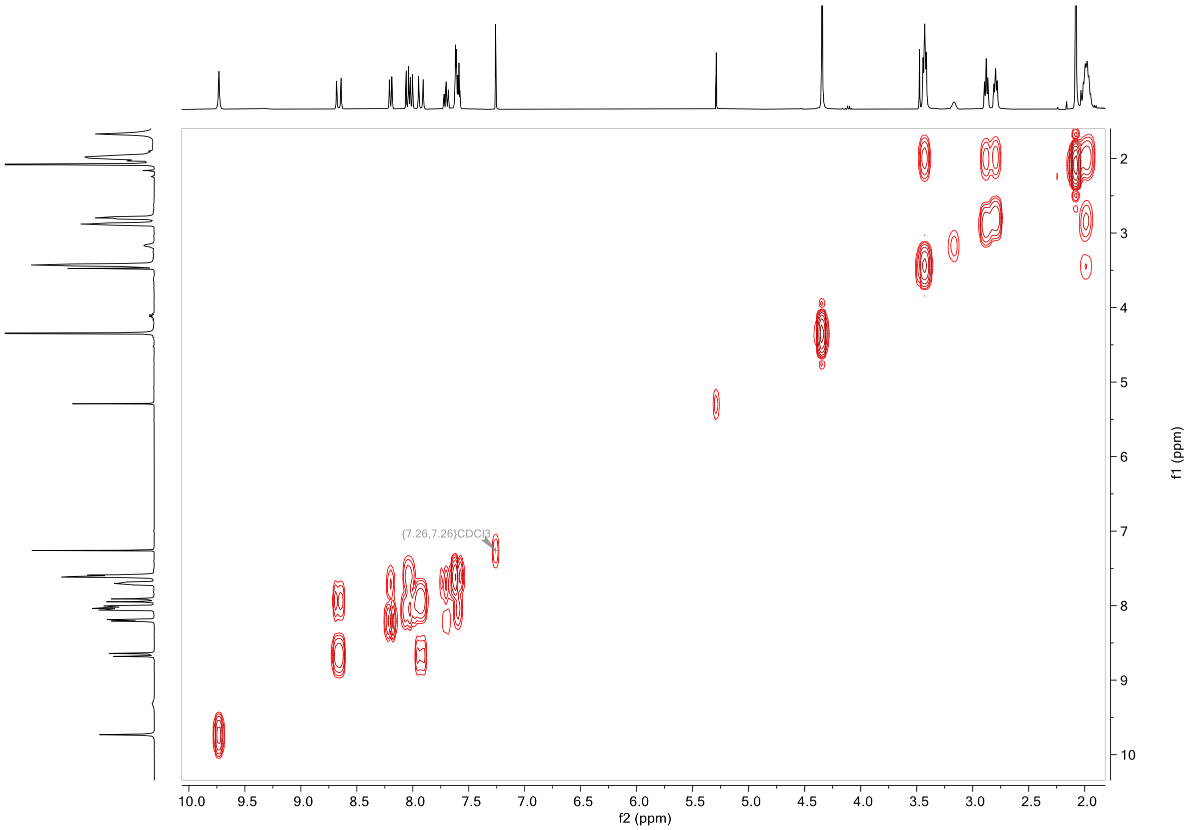

Figure S5. ^{1}H gCOSY NMR spectrum (400 MHz, $CDCl_3$) of **AztecM**.

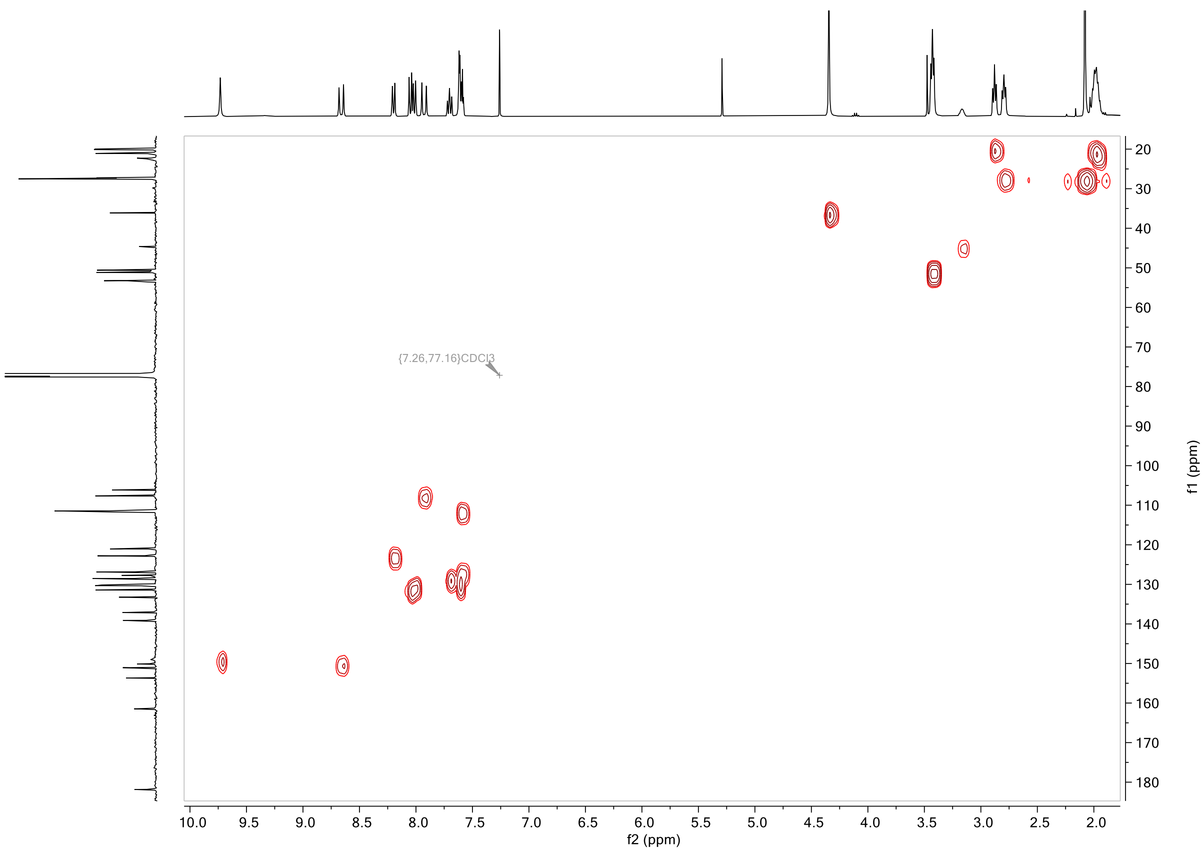

Figure S6. ^{1}H-^{13}C gHSQC NMR spectrum ($CDCl_3$, 400 MHz) of **AztecM**.

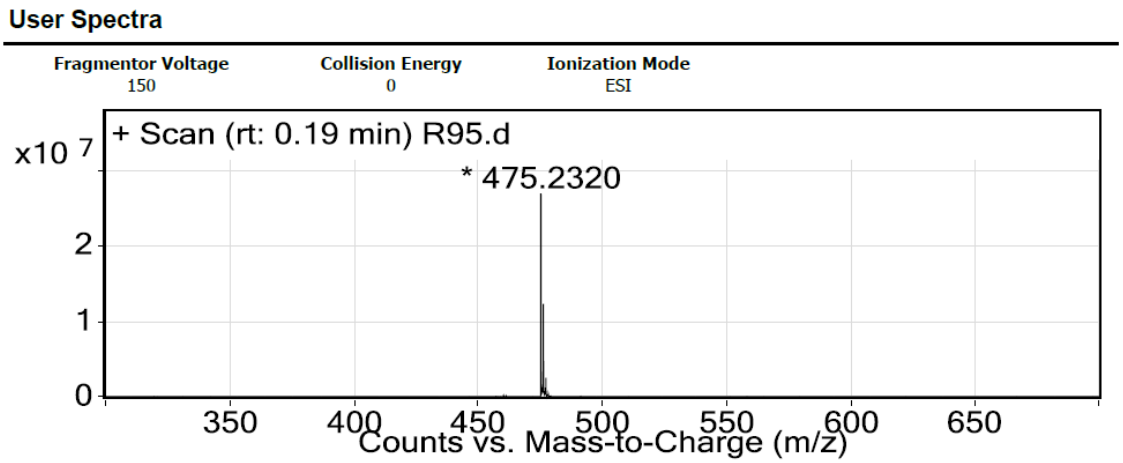

Figure S7. High-resolution mass ESI(+) spectrum of **AztecM**.

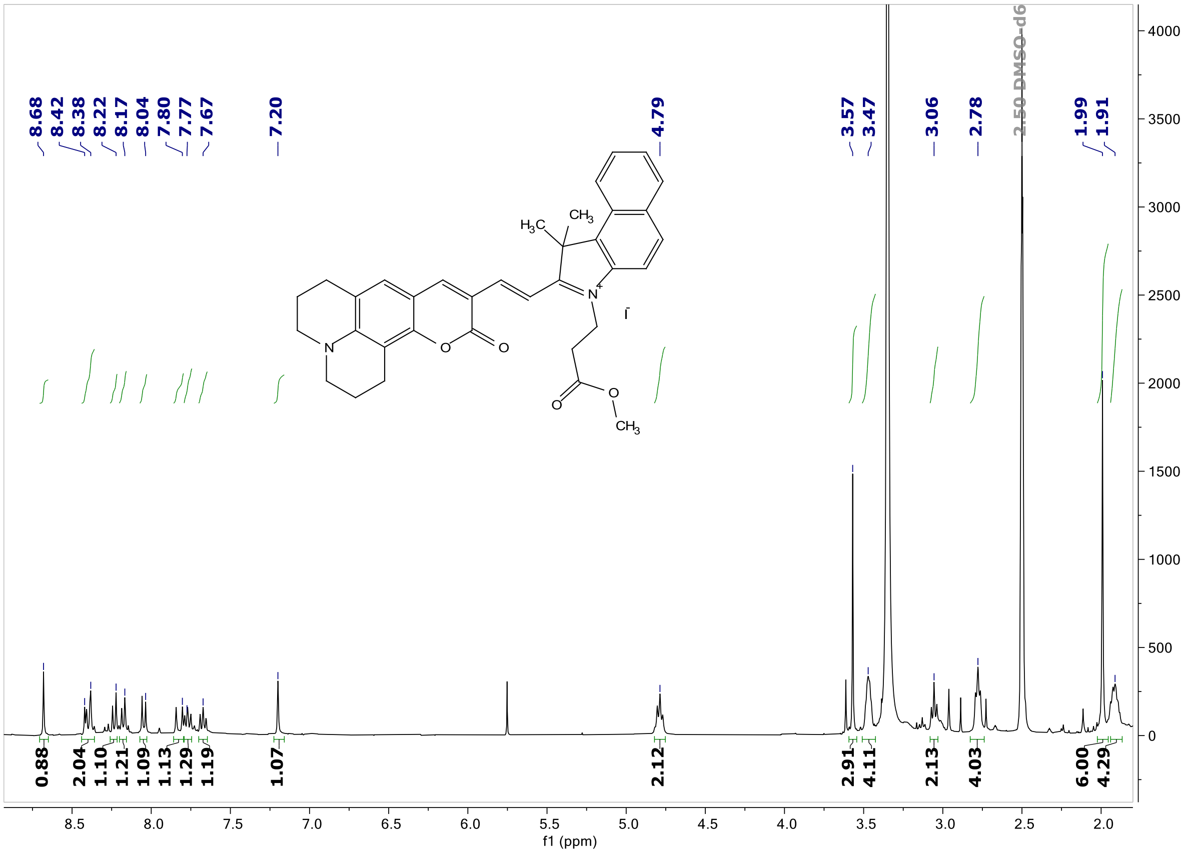

Figure S8. ^{1}H NMR spectrum (400 MHz, in DMSO-d_6) of **AztecM-LD**.

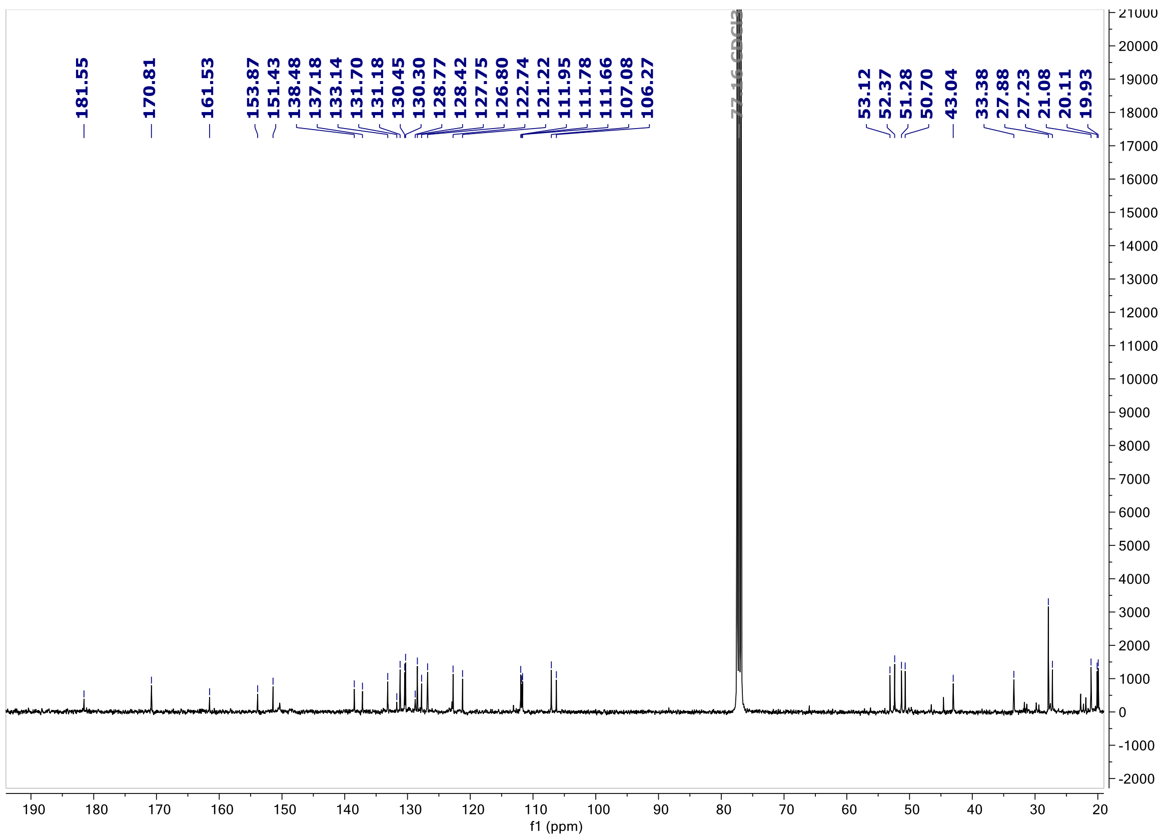

Figure S9. ^{13}C NMR spectrum (101 MHz, in $CDCl_3$) of **AztecM-LD**.

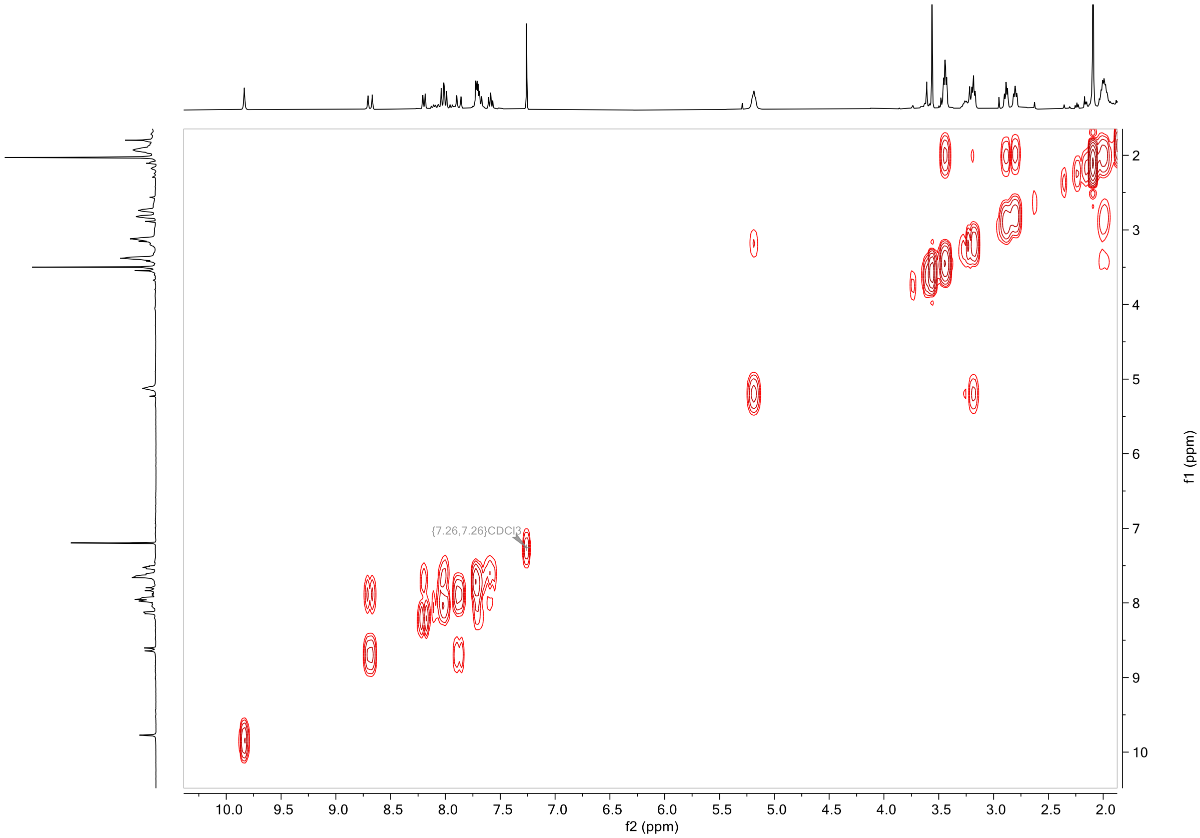

Figure S10. ^{1}H gCOSY NMR spectrum (400 MHz, $CDCl_3$) of **AztecM-LD**.

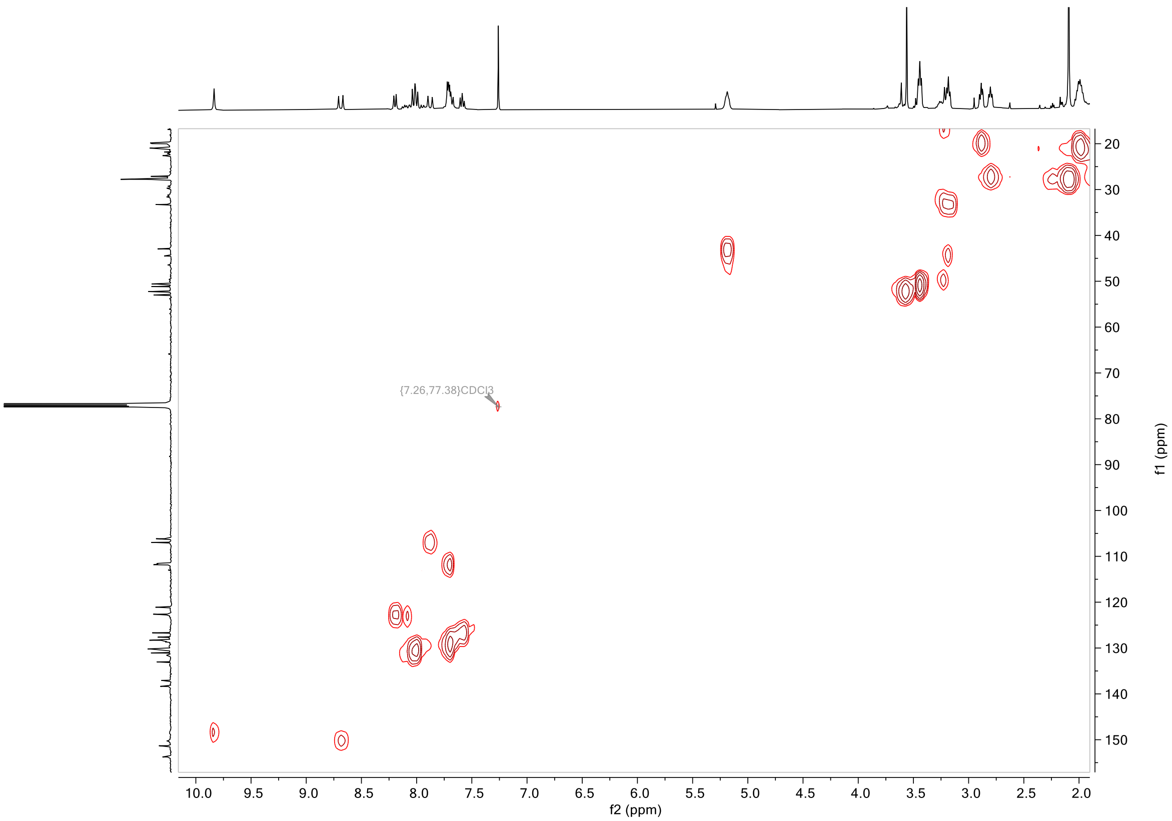

Figure S11. ^{1}H-^{13}C gHSQC NMR spectrum ($CDCl_3$, 400 MHz) of **AztecM-LD**.

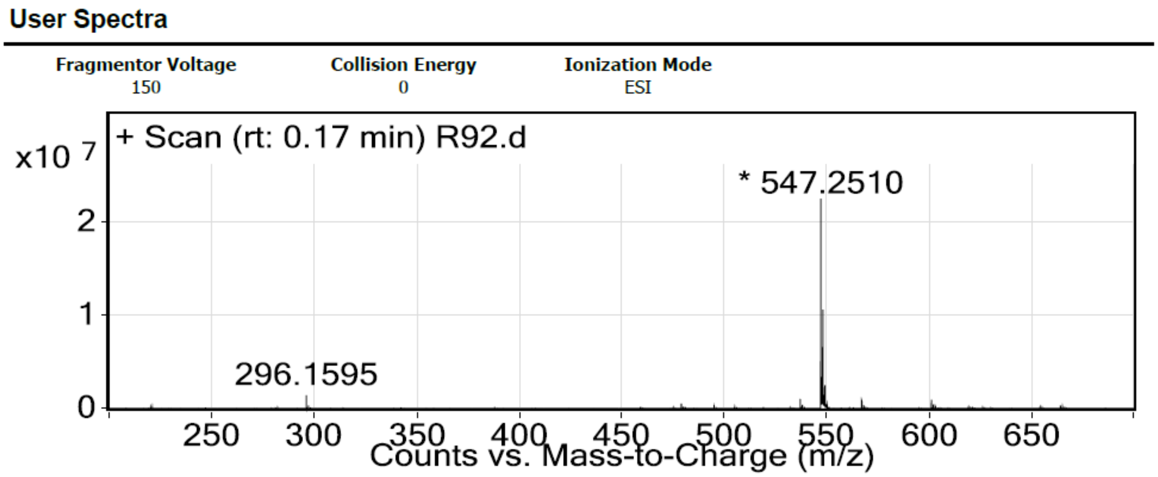

Figure S12. High-resolution mass ESI(+) spectra of **AztecM-LD**.

Synthesis of the spirocyclic AztecM-LD

The synthesis of **spirocyclic AztecM-LD** derivative was carried out following NaOH (1.2 eq, 480 mg, 12 mmol) to a solution of **AztecM-LD** (25 mg, 10 mmol) in ethanol (8 mL) during 2 hours at 40 °C. After that, the solvent was removed and sodium ethoxide in EtOH was added during 4 hours at 60 °C. Then, the crude product was vacuum-evaporated and recrystallized in cold ethyl acetate : hexane to afford the desired product quantitatively. A crystalline yellowish solid was obtained (final yield: 85%), however, the product was detected relatively to be relatively unstable in short periods of time as both, colour in solution turned brownish and crystallinity turned to an amorphous powder.

Spirocyclic AztecM-LD ^{1}H NMR (400 MHz, (DMSO-d_6) δ in ppm: 8.56 (s, 1H), 8.42-8.36 (m, 2H), 8.19 (dd, J = 15.70, 7.91 Hz, 2H), 8.00 (d, J = 8.75 Hz, 1H), 7.73-7.88 (m, 2H), 7.68 (t, J = 7.71 Hz, 1H), 7.38 (s, 1H), 4.63 (t, J = 6.90 Hz, 2H), 3.49 (s, 2H), 2.88-2.61 (m, 4H), 2.00 (s, 6H), 1.75 (q, J = 8.01, 6.42 Hz, 4H), 1.33-1.21 (m, 4H). ^{13}C NMR (101 MHz, $CDCl_3$) δ in ppm: 172.21, 163.15, 154.10, 152.34, 137.85, 137.74, 134.51, 132.21, 131.63, 130.98, 129.82, 128.95, 127.13, 126.85, 123.58, 122.74, 122.42, 113.07, 112.83, 112.69, 107.96, 106.45, 54.12, 53.35, 52.48, 51.86, 44.89, 43.24, 34.15, 28.31, 28.08, 23.42, 22.64, 21.87, 20.56.

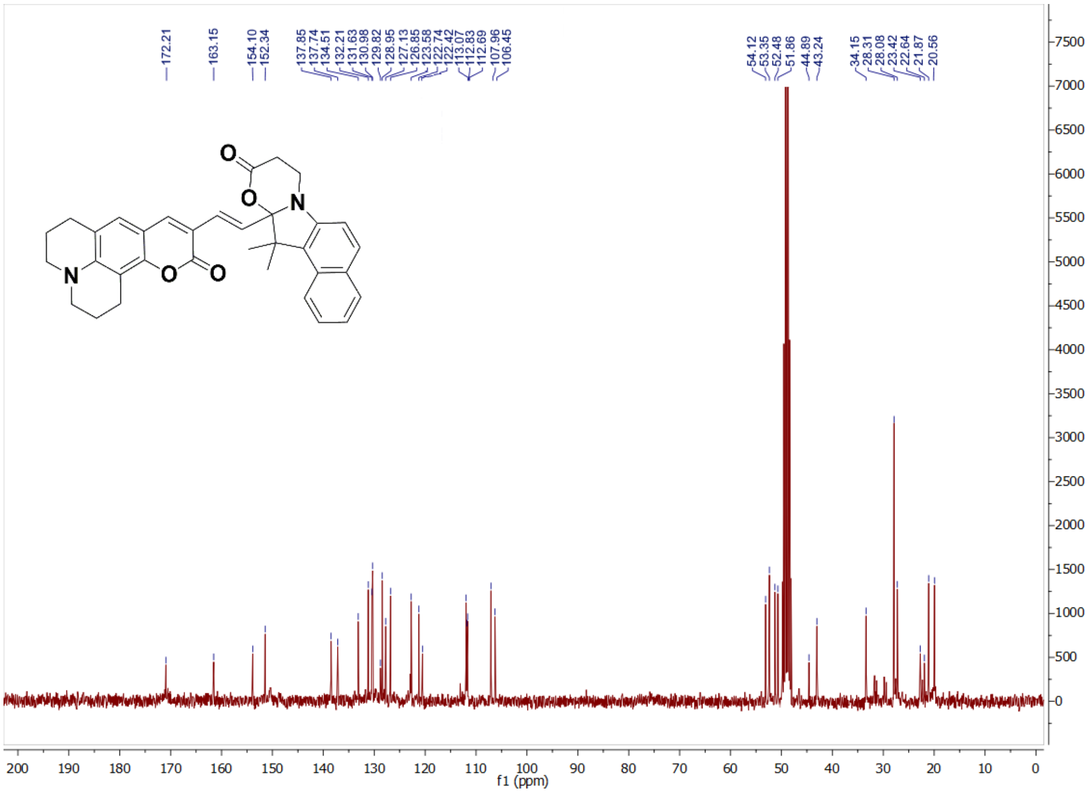

Figure S13. ^{13}C NMR spectrum (101 MHz, in DMSO-δ_6) of **spirocyclic AztecM-LD**.

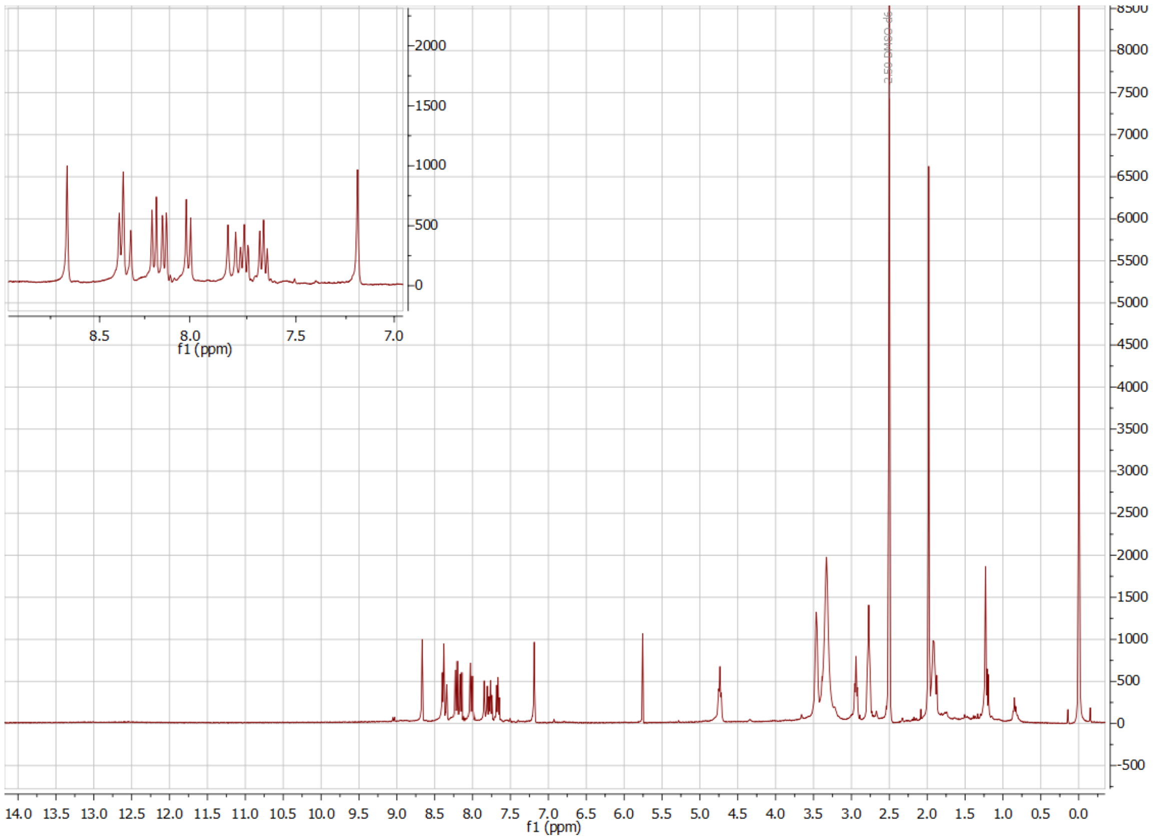

Figure S14. ^{1}H NMR spectrum (400 MHz, in DMSO-δ_6) of **spirocyclic AztecM-LD**.

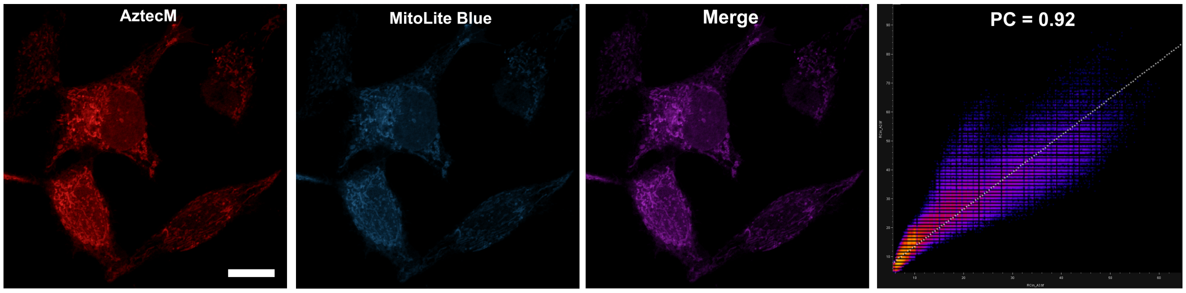

Figure S15. Co-localization imaging in live SK-Lu-1 cells for (A) synthesized spyrocyclic **AztecM** (red channel: λ_{exc} = 647 nm, λ_{em} = 700 nm) contrasted with MitoLite Blue lipid droplets co-localizer (blue channel, λ_{exc} = 410 nm, λ_{em} = 440 nm). The estimated Pearson's coefficient (PC) was calculated from scatter plot. To avoid blue signal contamination to the red channel, laser powers were maintained at 0.05 mW (0.2% from a 25 mW laser) and untreated cells were first recorded in order to subtract any native emission signal. Scale bars represent 20 μm.

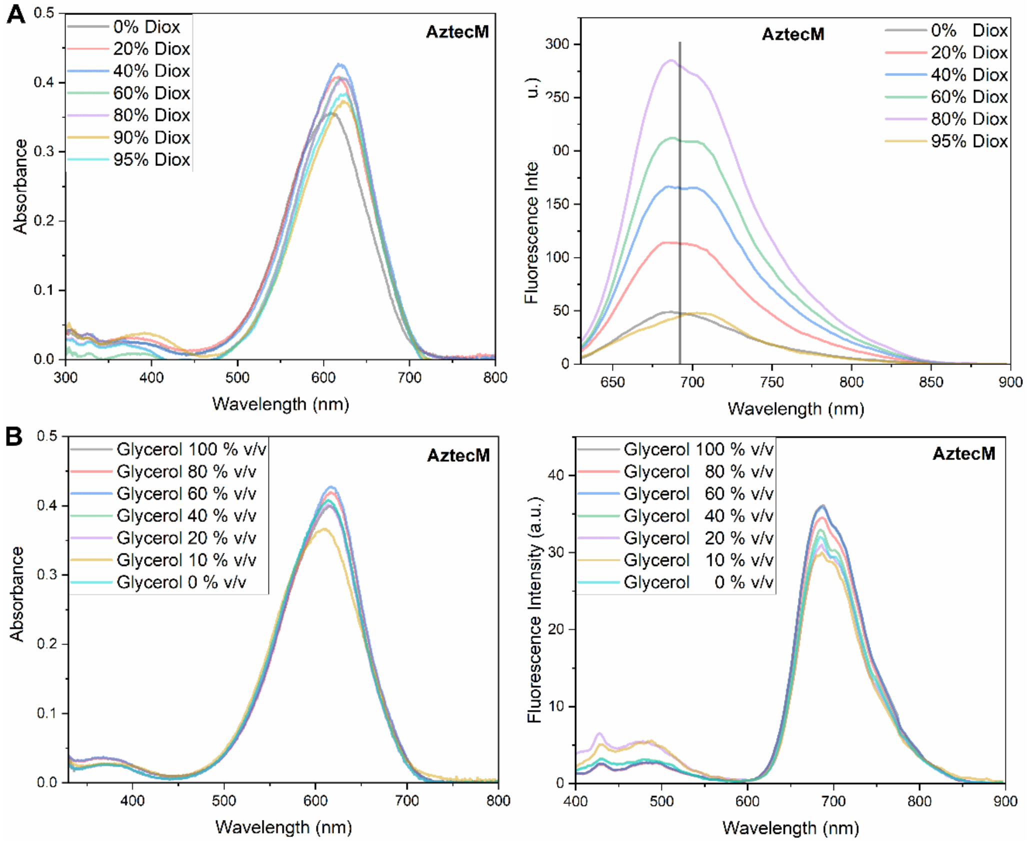

Figure S16. UV-Vis and fluorescence spectra of 3 mM **AztecM** recorded at (A) variable dioxane : H_2O volume mixtures to account for a dielectric constant variation from 12.22 [95% v/v Dioxane] to 80.40 [0% v/v Dioxane] and, (B) variable glycerol : methanol solvent mixtures to account for a viscosity variation window from 0.544 cP [0% v/v Glycerol] to 934 cP [100% v/v Dioxane].

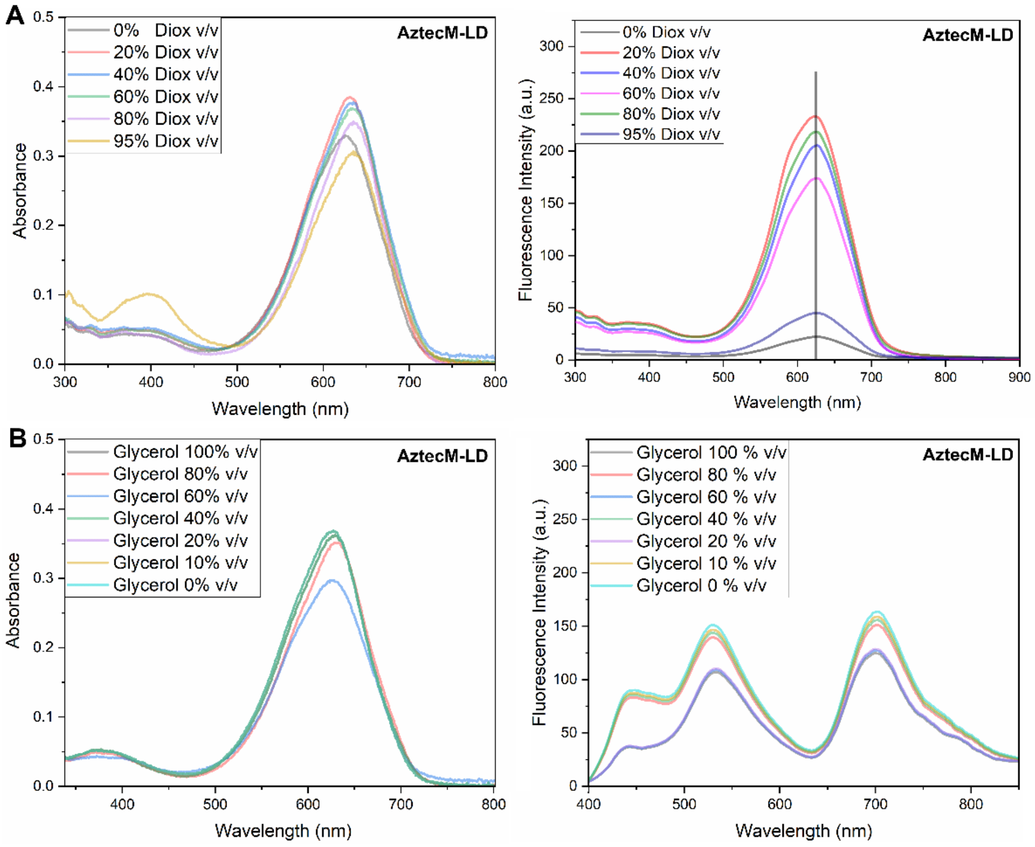

Figure S17. UV-Vis and fluorescence spectra of 3 mM **AztecM-LD** recorded at (A) variable dioxane : H_2O volume mixtures to account for a dielectric constant variation from 12.22 [95% v/v Dioxane] to 80.40 [0% v/v Dioxane] and, (B) variable glycerol : methanol solvent mixtures to account for a viscosity variation window from 0.544 cP [0% v/v Glycerol] to 934 cP [100% v/v Dioxane].

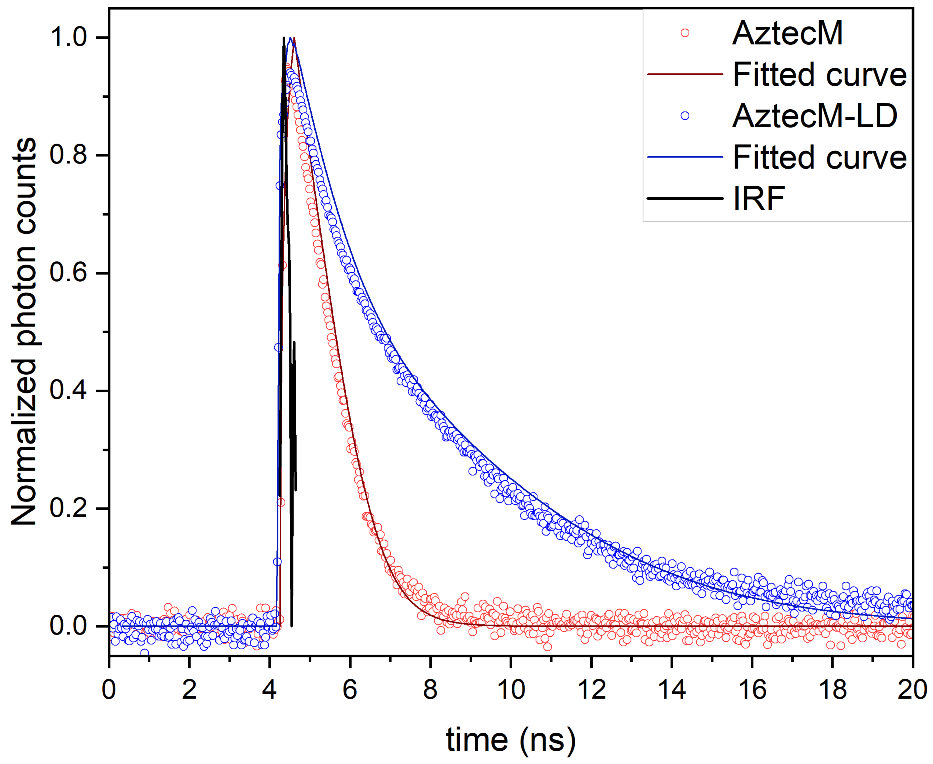

Figure S18. Time-Correlated Single-Photon Counting (TCSPC) decay curves for **AztecM** and **AztecM-LD**. The lifetimes were obtained in DMSO media (λ_{exc} = 480 nm) by a biexponential fitting to Equation S2: A + B1 × Exp($-t/\tau_1$) + B2 × Exp($-t/\tau_2$). Here τ_1 are presented in Table S1.

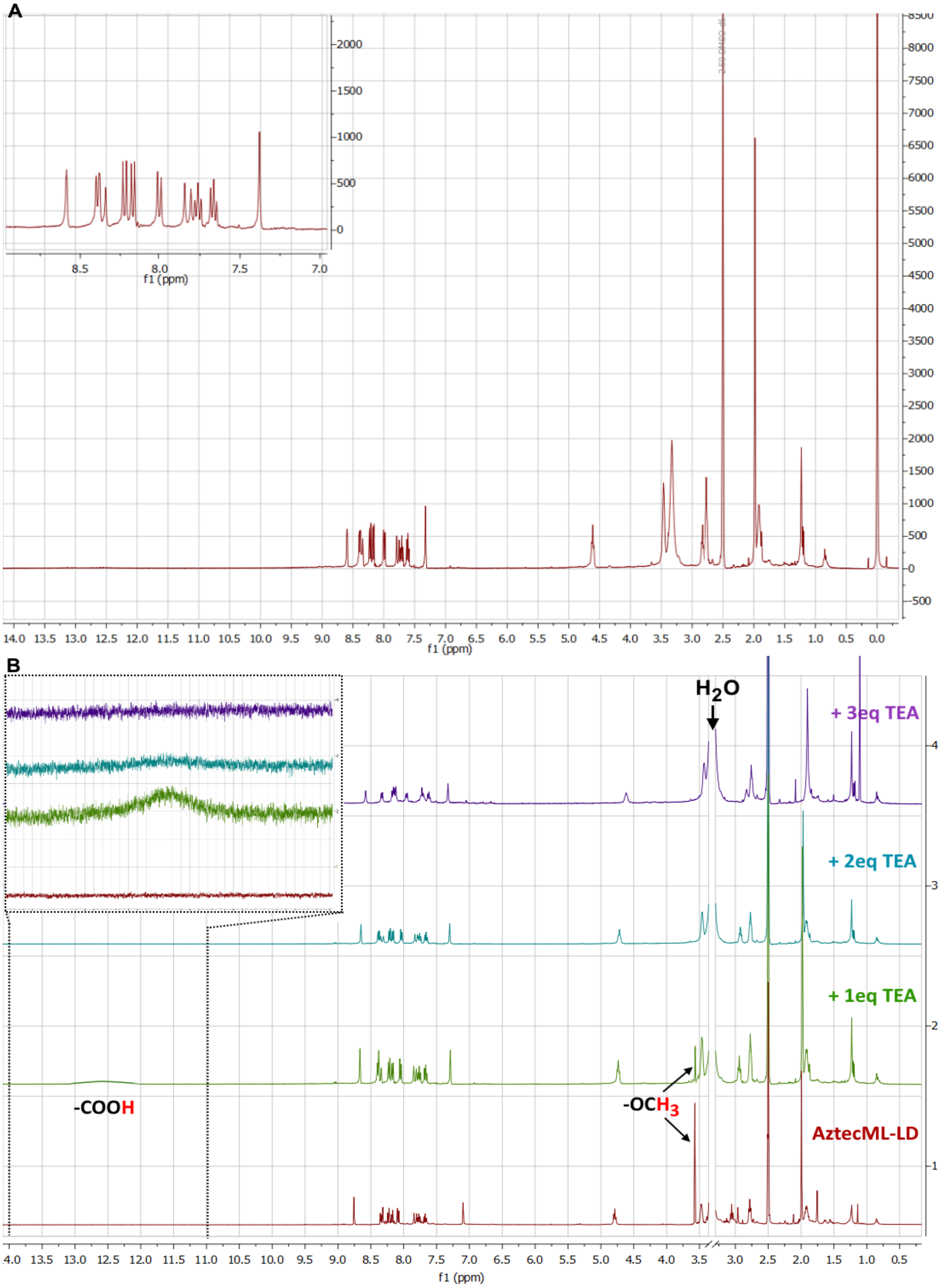

Figure S19. ^{1}H NMR spectra (400 MHz, DMSO-δ_6) for (A) synthetized **spirocyclic AztecM-LD** as described above and, (B) 0 to 3 equivalents of triethylamine (TEA) addition to the **AztecM-LD**.

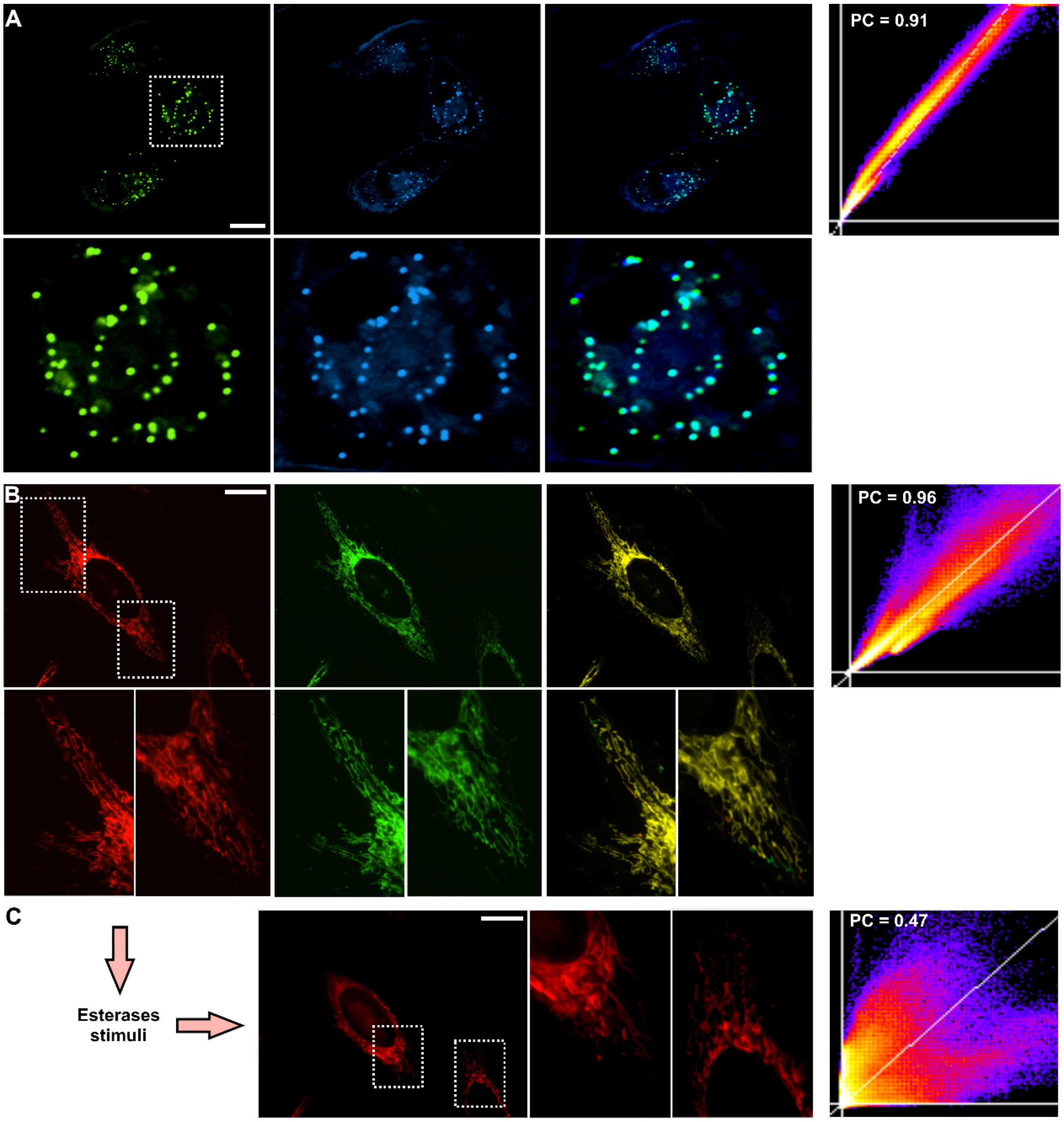

Figure S20. High-resolution confocal imaging using Airyscan technique for the co-localization in live SK-Lu-1 cells for (A) spyrocyclic **AztecM-LD** that was previously hydrolyzed (green channel: λ_{exc} = 488 nm, λ_{em} = 500 nm) contrasted with monodansylpentane (MHD, #SM1000a, Abcepta, San Diego, CA) lipid droplets co-localizer (blue channel, λ_{exc} = 410 nm, λ_{em} = 440 nm). To avoid MHD Blue signal contamination to the green channel, laser powers were maintained at 0.05 mW (0.2% from a 25 mW laser) and untreated cells were first recorded in order to subtract any native emission signal. (B) **AztecM-LD** (acetyl ester open form, red channel, λ_{exc} = 647 nm, λ_{em} = 700 nm) contrasted with MitoTracker Green (green channel: λ_{exc} = 488 nm, λ_{em} = 500 nm). The estimated Pearson's coefficients (PC) were calculated from scatter plots. (C) Effect of 5 μg/mL esterases stimuli for 2 hours incubation on the imaging profile of **AztecM-LD** (acetyl ester open form, red channel, λ_{exc} = 647 nm, λ_{em} = 700 nm, PC = 0.47). Scale bars represent 20 μm.

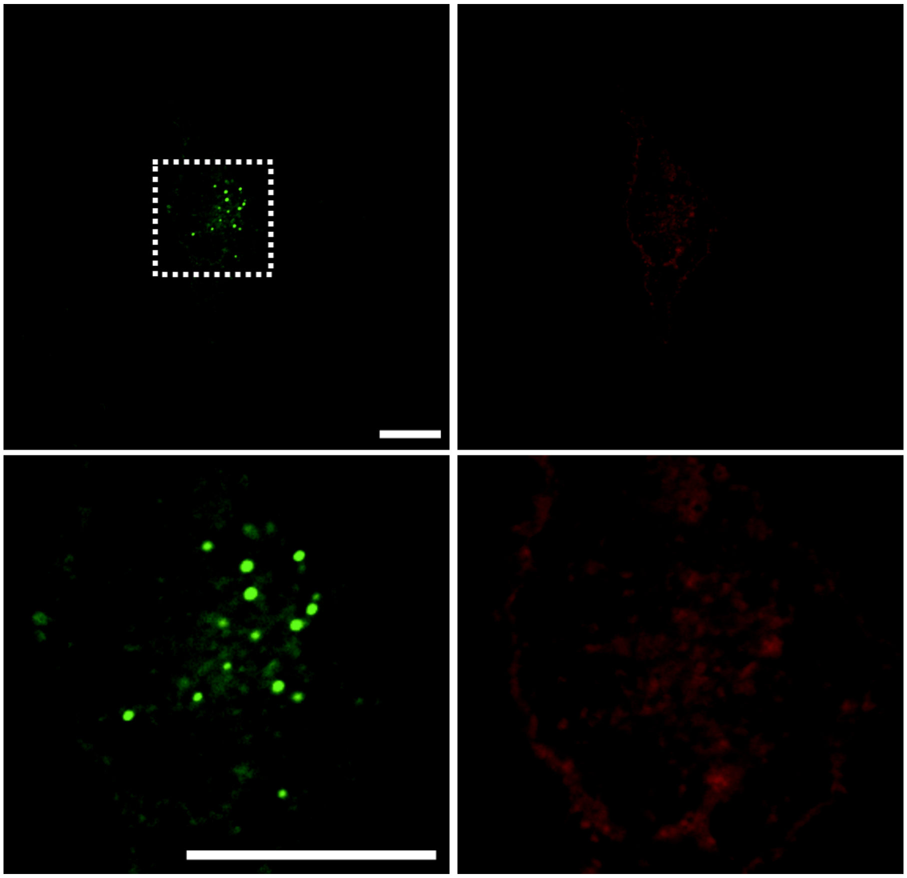

Figure S21. Imaging microscopy in live SK-Lu-1 cells for synthesized spyrocyclic **AztecM-LD** (green channel: λ_{exc} = 488 nm, λ_{em} = 500 nm; red channel, λ_{exc} = 647 nm, λ_{em} = 700 nm). Scale bars represent 20 μm.

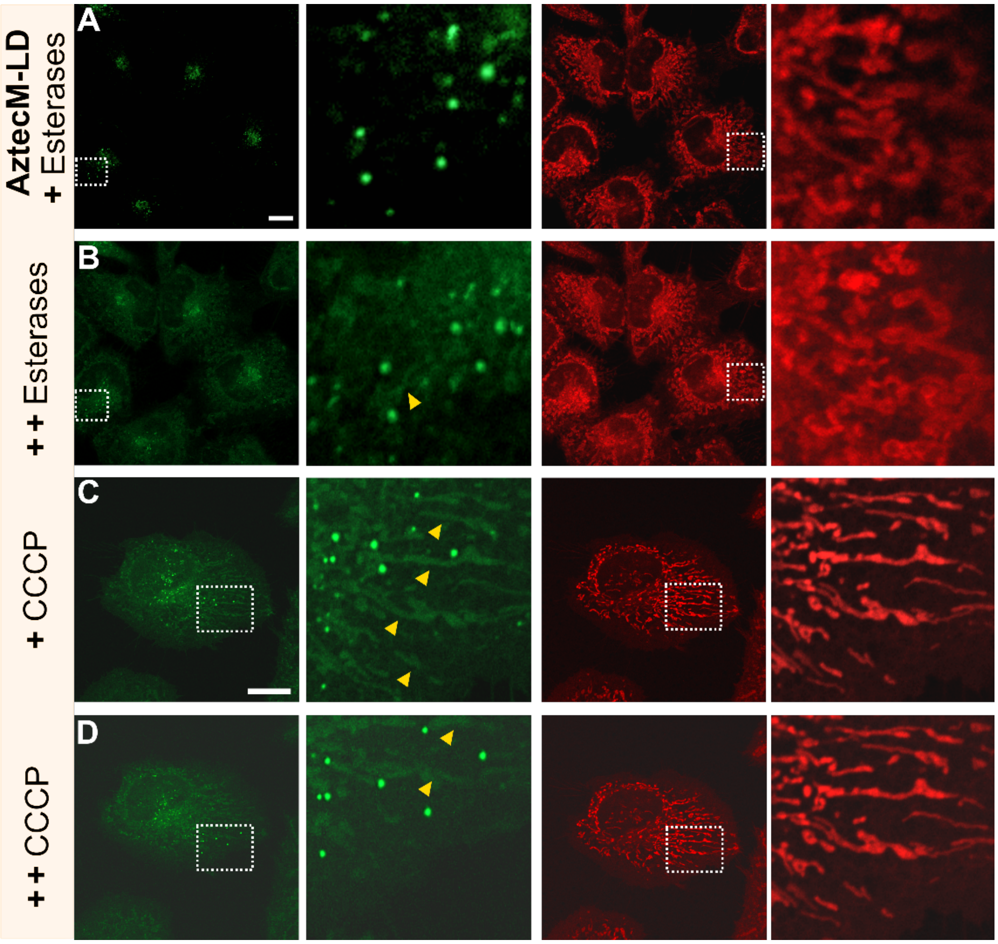

Figure S22. Confocal imaging in live SK-Lu-1 cells for the localization of 4 μM (A) **AztecM-LD** + 5 μg/mL esterases (B) further 5 μg/mL added after 15 min, (C) 10 μM CCCP and (D) further 10 μM CCCP *in situ* additions, detected at the green (λ_{em} = 500 nm) and red (λ_{em} = 710 nm) confocal channels. Scale bars represent 20 μm.

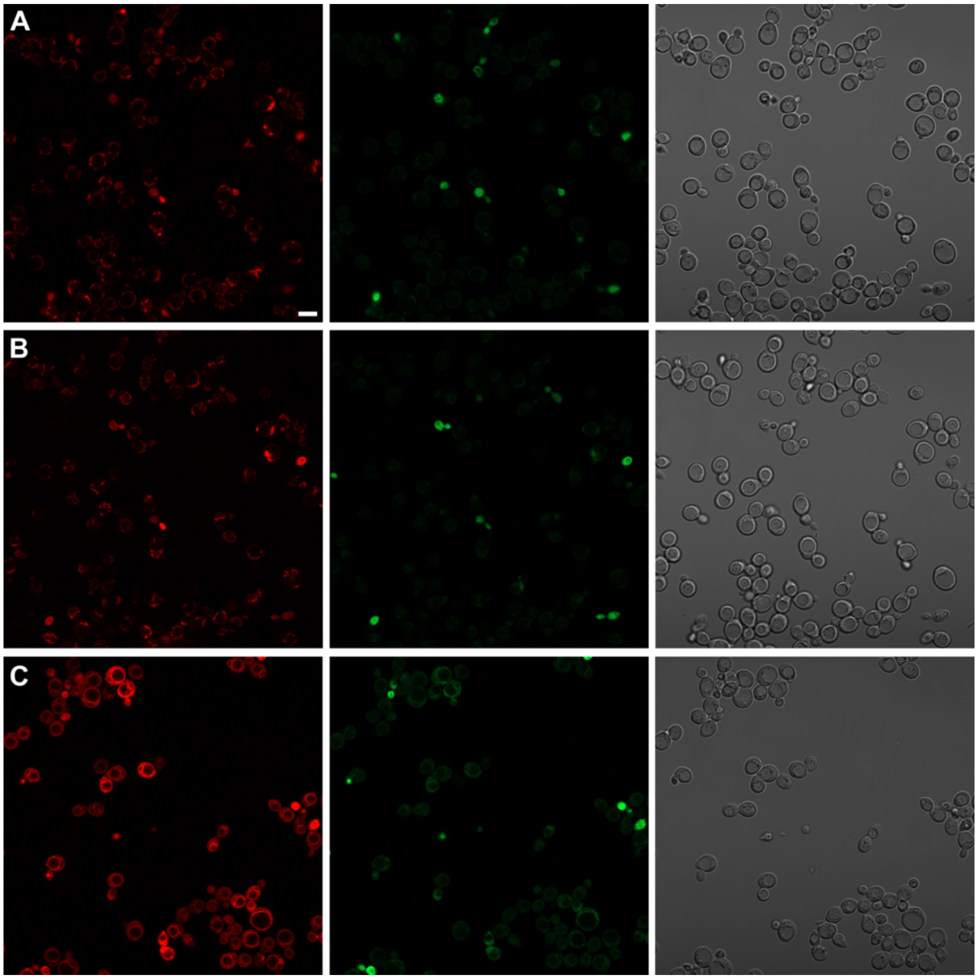

Figure S23. Fluorescence variation effect of *Saccharomyces cerevisiae* W303 yeast stained with (A) 2 μM **AztecM-LD**; (B) 10 μM CCCP uncoupler and (C) KCl stimuli. (A,B) Membrane localization and, (C) Fluorophore release by using KCl stimuli according to the conditions described in 'Experimental Methods' section. (Green: λ_{ex} = 488 nm, λ_{em} =500 nm) and (red: λ_{ex} = 647 nm, λ_{em} = 710 nm) confocal channels. Scale bars represent 5 μm.

Acknowledgements

Financial support by PAPIIT-UNAM IA200522 and Conahcyt grant no. PCC-319214 are gratefully acknowledged. C.H.-J. acknowledges Conahcyt grant CVU 771165. We also acknowledge the assistance of Ruth Rincón Heredia (PhD. Unidad de Imagenología del IFC-UNAM) in imaging microscopy, Adriana Romo Pérez, Ph.D. and Teresa Ramírez Apán, M.Sc. in tissue culture and Everardo Tapia Mendoza (Ph.D. in HRMS studies), Elizabeth Huerta Salazar, M.Sc. in NMR and Beatriz Quiróz García, (PhD. NMR lab: LURMN at IQ-UNAM) which is funded by CONACYT Mexico (0224747), and UNAM”.
